# Gainers and losers of surface and terrestrial water resources in China during 1989–2016

**DOI:** 10.1038/s41467-020-17103-w

**Published:** 2020-07-10

**Authors:** Xinxin Wang, Xiangming Xiao, Zhenhua Zou, Jinwei Dong, Yuanwei Qin, Russell B. Doughty, Michael A. Menarguez, Bangqian Chen, Junbang Wang, Hui Ye, Jun Ma, Qiaoyan Zhong, Bin Zhao, Bo Li

**Affiliations:** 1 Coastal Ecosystems Research Station of the Yangtze River Estuary, Ministry of Education Key Laboratory for Biodiversity Science and Ecological Engineering, Institute of Biodiversity Science, School of Life Sciences, Fudan University, Shanghai, 200438 China; 2 Department of Microbiology and Plant Biology, Center for Spatial Analysis, University of Oklahoma, Norman, OK 73019 USA; 3 Department of Geographical Sciences, University of Maryland, College Park, MD 20742 USA; 4 Key Laboratory of Land Surface Pattern and Simulation, Institute of Geographic Sciences and Natural Resources Research, Chinese Academy of Sciences, Beijing, 100101 China; 5 LinkedIn Corporation, Sunnyvale, CA 94085 USA; 6 Rubber Research Institute, Chinese Academy of Tropical Agricultural Sciences, 571737 Danzhou, Hainan China; 7 Key Laboratory of Ecosystem Network Observation and Modeling, Institute of Geographic Sciences and Natural Resources Research, Chinese Academy of Sciences, Beijing, 100101 China

**Keywords:** Hydrology, Freshwater ecology, Water resources

## Abstract

Data and knowledge of the spatial-temporal dynamics of surface water area (SWA) and terrestrial water storage (TWS) in China are critical for sustainable management of water resources but remain very limited. Here we report annual maps of surface water bodies in China during 1989–2016 at 30m spatial resolution. We find that SWA decreases in water-poor northern China but increases in water-rich southern China during 1989–2016. Our results also reveal the spatial-temporal divergence and consistency between TWS and SWA during 2002–2016. In North China, extensive and continued losses of TWS, together with small to moderate changes of SWA, indicate long-term water stress in the region. Approximately 569 million people live in those areas with deceasing SWA or TWS trends in 2015. Our data set and the findings from this study could be used to support the government and the public to address increasing challenges of water resources and security in China.

## Abstract (web summary)

The authors of this study compile data on spatial and temporal dynamics of surface water bodies across China, covering a time span from 1989 – 2016. The study describes hot-spot areas with strongly decreasing trends in surface water area and terrestrial water storage in North China and discusses implications of water resources and security in China.

## Introduction

Surface water resources are important for aquatic and terrestrial ecosystems, agriculture, industry, and societies across local, national, and global scales^1–3^. China is the most populous country in the world, and its gross domestic product (GDP) has increased at an average rate of 10% for more than 20 years^4^. However, the sustainability of the economic growth and environmental security of the country are increasingly threatened by environmental degradation and resource constraints, particularly water resources^4,5^. Some parts of China have abundant water resources, but other regions are naturally arid and semi-arid climate and have very limited surface water resource, such as North China^4,6^. Furthermore, China has frequently suffered from floods and droughts^7,8^. Therefore, accurate and updated information on the spatial–temporal dynamics of surface water bodies over the past few decades across different spatial scales are invaluable for better understanding of the roles of surface water bodies in water security in China, but such information has not been well documented to the public and policy-makers, yet.

A number of surface water body data sets at moderate to coarse spatial resolutions have been generated and used to monitor surface water bodies, such as the downscaled GIEMS-D15 at 15 arc-s for 1993–2007^9^, the Global Water Body data (GLOWABO) at 0.5 arc-s resolution in 2000^10^, the 1-km Global Lake and Wetland Database (GLWD) in 2000^11^, and the global monthly surface water extent at 0.25° for 1993–2007^12^. However, the spatial resolutions of these data sets are not high enough (tens of meters) to identify small inland surface water bodies^13^.

Satellite-based remote sensing images at high spatial resolutions (tens of meters) have been widely used to map surface water bodies^14–16^. After Landsat data (30-m spatial resolution) became freely available and open access in 2008^17^, several studies have reported changes in surface water area (SWA) using Landsat images in China^18–22^. However, some of these studies used images for specific dates in different years for comparison^18–20^, which might lead to very different results and temporal uncertainties in SWA due to the strong seasonal dynamics and interannual variation of surface water bodies^23^, and some of these studies only focused on certain hotspots in China^21,22^. Furthermore, several studies have reported the area changes of only lakes, ponds, or reservoirs^13,24–26^, but did not include other surface water bodies such as rivers and streams.

Several global surface water maps have been generated by analyzing Landsat imagery, such as the 30-m GLCF Inland Surface Water data set (GIW) in 2000^10^ and the global 90-m surface water body map^14^. These maps were generated by analyzing Landsat images acquired in specific year(s) and could hardly be used to document annual and multi-decadal changes of surface water bodies in China. Recently, the global surface water body data set released by the Joint Research Centre (JRC) in 2016 reported the monthly dynamics of permanent and seasonal surface water bodies at 30-m resolution from 1984 to 2015^2^. However, the data set missed most of the surface water bodies in 1994 for China, and failed to detect the large floods in South China during 1997–1998 (Supplementary Fig. 1), which was considered the worst flood event in the past 40 years in South China and resulted in an economic loss of US$20 billion^7^. In addition, the JRC data set used the Landsat top-of-atmosphere reflectance data (no atmospheric correction) as input data, which might introduce some uncertainties into the resultant maps^27^. The JRC data set used many ancillary maps and data products that may reduce the commission error of maps, but this procedure makes the method more complex and the resultant maps are affected by those inaccuracies and uncertainties of the ancillary maps and data sets.

Surface water is one of the major components of the terrestrial water storage (TWS). The global TWS is estimated to have surface water (36.08 ± 9.89%), groundwater (37.56 ± 16.57%), soil water (26.36 ± 7.46%), and others (vegetation water, snow, and ice)^28^. The changes in SWA have substantial effects on the dynamics of TWS^29^. For example, the expansion of surface water bodies can recharge groundwater and TWS, and the shrinkage of surface water bodies can cause a large decrease of TWS and hence more groundwater was used^27,30^. Thus, quantifying the relationship between SWA and TWS could provide valuable information about the roles of SWA and groundwater in the spatial–temporal dynamics of TWS, and could also help us better understand the effects of floods and droughts on water resources and the feedbacks between surface water bodies and TWS. To date, there have been only a few regional-scale studies in China^29^ and the spatial–temporal relationship between SWA and TWS at different scales have not been quantified. Both SWA and TWS can be affected by climate and anthropogenic activities, such as precipitation^23,27^, temperature^23,27^, dam construction^31^, consumptive use, and agriculture irrigation^18,27^. To date, there have been many local-scale studies in China^21,22,32^, but the overall information about the effects of climate and anthropogenic activities on the spatial–temporal variation of SWA and TWS in China has not yet been fully investigated.

Here, we first use a simple and robust surface water mapping algorithm^21,23,27,32^ and all the available Landsat TM/ETM+/OLI surface reflectance images in the Google Earth Engine (GEE) cloud computing platform and generate annual maps of surface water bodies in China from 1984 to 2016 at 30-m spatial resolution. We report annual maps of surface water bodies in China during 1989–2016, as Landsat image numbers during 1984–1988 in China are small (Supplementary Fig. 12c). Second, we quantify the spatial–temporal dynamics of SWA and TWS at different spatial scales during 1989–2016, and investigate the spatial–temporal relationships between SWA and TWS during 2002–2016. Third, we investigate the effects of climate and anthropogenic activities (e.g. new dam construction and reservoirs, water transfer projects, and human water use) on SWA and TWS. Finally, we assess the changes in human population in relation to changes in SWA and TWS, which identify the hotspots where people have already experienced losses of SWA and TWS.

## Results and discussion

### Surface water frequency maps and surface water areas during 1989–2016

**Fig. 1:**
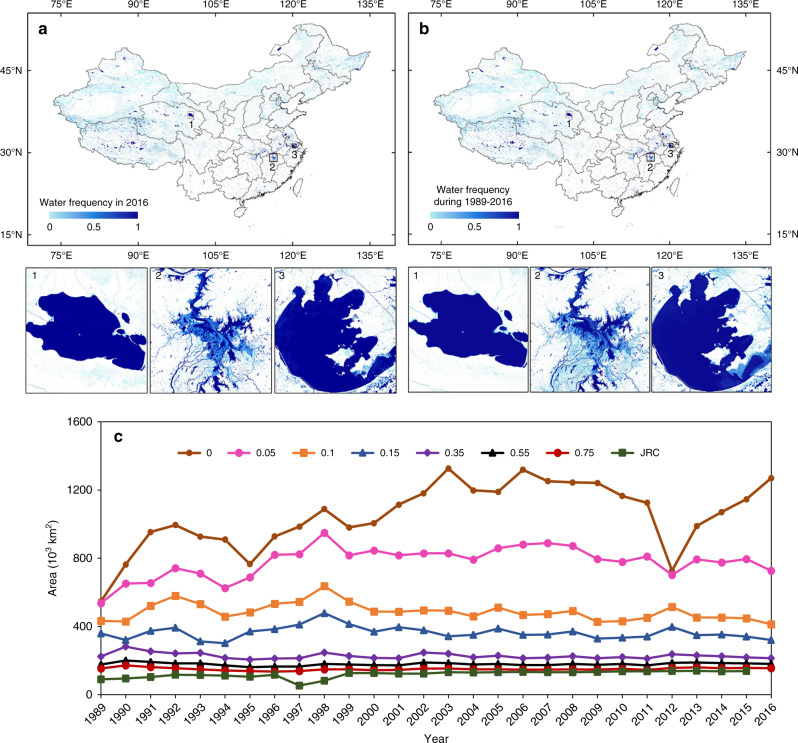
Surface water body frequency maps and surface water areas using different frequency thresholds in China. **a** Surface water body frequency map in 2016 for China, and (1, 2, 3) are its three zoom-in views for Qinghai Lake, Poyang Lake, and Taihu Lake, respectively. **b** The 28-year surface water body frequency map during 1989–2016. **c** Water body areas within China during 1989–2016 with different annual water frequencies. The permanent water body areas from the Joint Research Centre (JRC) data set^2^ during 1989–2015 are also shown.

Surface water frequencies (FW) of individual pixels in 2016 varied substantially across China (Fig. 1a). There were 1444 million pixels with annual surface water frequency of FW > 0 in 2016, amounting to ~1.3 × 10^6^ km^2^ maximum SWA in 2016. Based on the surface water frequency in a year, a water pixel was defined as year-long surface water (FW ≥ 0.75), seasonal surface water (0.05 ≤ FW < 0.75) or ephemeral surface water (FW < 0.05)^21,23,27,32^. The year-long SWA in 2016 was ∼0.155 × 10^6^ km^2^, most of which was in large rivers, lakes, and reserves (Fig. 1a) between 80°–90°E and 110°–120°E longitude and 35°–45°N latitude in China (Supplementary Fig. 2a). The seasonal and ephemeral surface water areas in 2016 were 0.571 × 10^6^ and 0.542 × 10^6^ km^2^, respectively, most of which were located at the edge of large surface water bodies, small ponds and streams, and flooded rice fields (Fig. 1a). The 28-year surface water frequency of individual pixels during 1989–2016 also had large spatial variation in China (Fig. 1b). There were 181 million pixels with 28-year surface water frequency of FW ≥ 0.75, amounting to 0.163 × 10^6^ km^2^ SWA, which was 5% higher than that in 2016. The spatial distribution of annual surface water frequency map in 2016 agreed statistically well with that of 28-year surface water frequency map (Supplementary Fig. 3), but they did have notable differences, which might be related to climate, dam and reservoir construction, water management, and water use.

At the national scale, we calculated SWA in a year with various surface water frequencies, ranging from FW > 0 to FW ≥ 0.75 (Fig. 1c). The year-long SWA (FW ≥ 0.75) in China varied from 0.135 × 10^6^ km^2^ in 1996 to 0.172 × 10^6^ km^2^ in 1990 over the period of 1989–2016 (Fig. 1c), and it had the smallest standard deviation and extremum (Supplementary Fig. 4). The year-long SWA in China increased significantly during 1991–2016 (slope = 0.36 ± 0.33 × 10^3^ km^2^ year^−1^, *p* < 0.05). The seasonal SWA (0.05 ≤ FW < 0.75) had large increases during 1989–1998 and then remained relatively stable during 1999–2016 (Fig. 1c). The ephemeral SWA (0 < FW < 0.05) had large increases during 1989–2003 and then remained stable during 2004–2016 (Fig. 1c), and the large drop in ephemeral SWA in 2012 was related to drought^33^ and the smaller number of good-quality Landsat observations (Supplementary Fig. 12b).

We compared the year-long SWA in China from our data set with the permanent SWA from the JRC data set^2^ (Fig. 1c). The JRC data set, which reported the permanent and seasonal surface water areas at 30-m resolution in the world from 1984 to 2015^2^, represents significant progress in remote sensing of surface water. In 2015, the year-long SWA in China from our data set (0.157 × 10^6^ km^2^) was moderately (12.9%) higher than the permanent SWA from the JRC data set (0.139 × 10^6^ km^2^). Over the period of 1989–2015, the year-long SWA in China in our data set agreed well with permanent SWA from the JRC data set (slope = 0.98 ± 1.1 × 10^3^ km^2^ year^−1^, *R*^2^ = 0.99, standard error = 0.56 × 10^3^ km^2^, *N* = 832), except 1997 and 1998 (Fig. 1c; Supplementary Fig. 5). The permanent and seasonal surface water areas in South China in 1997 and 1998 from the JRC data set were substantially lower than those from our data set (Supplementary Figs. 1 and 5), which raises concern on the use of the JRC data set for the study of extreme flood events in South China in 1998^7^. The differences in SWA estimates between our data set and the JRC data set can be attributed to the definition of surface water types (year-long vs permanent surface water), number of Landsat images used, Landsat image data types (top-of-atmosphere reflectance vs surface reflectance), training and validation data, and mapping algorithms. The JRC data set used the Landsat top-of-atmosphere reflectance images as data resources, many ancillary maps from other sources as masks, and 40,124 points for accuracy assessment of the global surface water maps. In this study, we used Landsat surface reflectance images, and 18,397 points for accuracy assessment of the surface water maps of China. The accuracy assessment showed that the user’s accuracies of our surface water body maps (year-long surface water: 99.7% (±0.12), seasonal surface water: 98.6% (±0.47)) are similar to those from the JRC data set, but the producer’s accuracy of seasonal surface water of our data set (86.4% (±3.57)) was higher than that of the JRC data set (68.4%) (Supplementary Table 4). Therefore, our surface water data set provides improved and reliable information about the surface water bodies in China during 1989–2016.

### Spatial–temporal dynamics of year-long SWA during 1989–2016

**Fig. 2:**
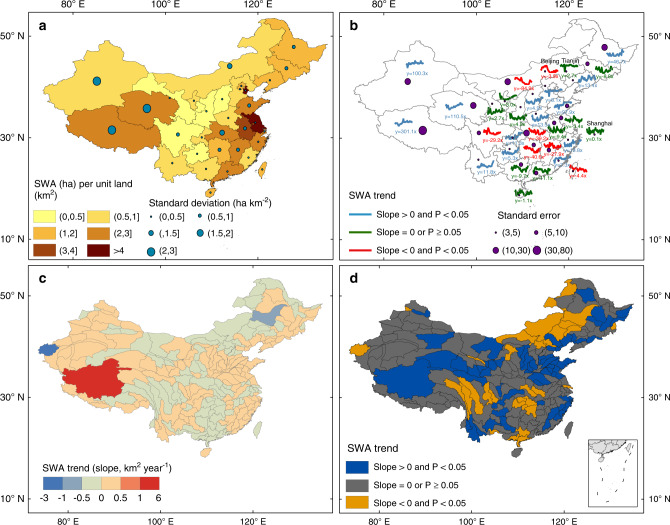
Interannual variations and trends of year-long surface water area during 1989–2016 by province and watershed in China. **a** Average year-long surface water area (SWA) (ha) per unit land area (km^2^) and standard deviation at the provincial scale. **b** Interannual trends of year-long SWA and standard errors at the provincial scale. **c** Interannual trends of year-long SWA at the watershed scale (slope value). **d** Interannual trends of year-long SWA at the watershed scale. Note that in 1996 SWA values in Tianjin, Hebei, and Shandong were extremely low (**b**), which was partially attributed to severe drought in the year. We analyzed the trend during 1989–2016 with 1996 data and without 1996 data, the slope values of the trend varied slightly in these three places. Here we keep the entire time series data in the graph, but only using the data without 1996 for regression model.

Year-long SWA at the provincial level was unevenly distributed across China with various interannual variations (Fig. 2a). The mean of year-long SWA (ha) per unit land area (km^2^) in a province during 1989–2016 varied between 0.16 ha km^−^^2^ in Gansu and Guizhou and 9.5 ha km^−^^2^ in Jiangsu. The standard deviation of year-long SWA per unit land area in a province ranged from 0.01 ha km^−2^ in Shanghai to 7.0 ha km^−^^2^ in Tibet. All the provinces in the Loess Plateau, the Mongolia Plateau, the Yunan-Guizhou Plateau, and mountainous areas (Fujian, Guangxi) had <1 ha km^−^^2^ SWA (Fig. 2a). Hebei and Henan Provinces also had <1 ha km^−^^2^ SWA, where annual precipitation was moderate and unevenly distributed^34,35^ and surface and groundwater withdrawal for public water supply and irrigation substantially increased^34,36^. Xinjiang had <1 ha km^−^^2^ SWA because of its arid climate and large land area. Three provinces in Northeast China had 1–2 ha km^−^^2^ SWA. Provinces in the Qinghai-Tibetan Plateau had 2–3 ha km^−^^2^ SWA because of its large number of lakes and increased precipitation and glacial meltwater^20,37–39^. Provinces in East China and Southeast China are associated with the lower streams of Yellow River (Shandong), Yangtze River (Hubei, Jiangxi, Anhui, Jiangsu), Pearl River (Guangdong), and large lakes (Jiangxi), and thus had 2–3 ha km^−^^2^ or higher SWA. Overall, Southwest and Southeast China had much more SWA than other regions, especially North China, which is similar to the spatial patterns of annual precipitation in China^35^.

The year-long SWA in a province had divergent interannual trends during 1989–2016 in China (Fig. 2b). Fourteen provinces had significantly increasing trends of the year-long SWA during 1989–2016, ranging from 4.9 ± 1.5 km^2^ year^−^^1^ in Hebei to 301.1 ± 140.7 km^2^ year^−^^1^ in Tibet. Qinghai Province ranked the second in its increase of SWA (110.5 ± 52.4 km^2^ year^−^^1^). Among the provinces under arid and semi-arid climate, Xinjiang was the only province with significant increase of SWA (100.0 ± 68.4 km^2^ year^−^^1^). Increased annual precipitation and water from melting glaciers resulted in an increase of year-long SWA in Xinjiang Province^37^. In contrast, seven provinces had significantly decreasing trends of the year-long SWA during 1989–2016, ranging from −3.9 ± 1.7 km^2^ year^−^^1^ in Beijing to −84.9 ± 32.0 km^2^ year^−^^1^ in Inner Mongolia. The year-long SWA in Inner Mongolia shrank from 4660.6 km^2^ in 1991 to 3071.4 km^2^ in 2009, a loss of 1589.2 km^2^ or 34.1%^32^. The coal mining industry is one of major reasons for the large loss of lakes in Inner Mongolia, as the number of mining enterprises in Inner Mongolia increased markedly from 156 in 2000 to 865 in 2010 and annual coal production increased from 72 to 789 million tons^18^.

The year-long SWA in a watershed also had divergent interannual trends during 1989–2016 (Fig. 2c, d). Sixty-one watersheds, mostly in the western and northern Tibetan Plateau, had significantly increasing trends of year-long SWA during 1989–2016, ranging from 0.004 ± 0.001 km^2^ year^−^^1^ in the Qindanhe Watershed in Shanxi to 6.5 ± 1.1 km^2^ year^−^^1^ in the Qiangtang Plateau watershed in western Tibet. Water from melting glaciers and increased annual precipitation over the recent decades were considered as the major driving factors for the expansion of large amounts of lakes in the Tibetan Plateau^20,37–39^. Annual precipitation increased by 20 mm and annual mean air temperature increased by 1.6 °C from 2000 to 2014^40^. In addition, successful water conservation through the Chinese Ecological Protection and Construction Projects also contributed to the increasing trends of SWA in eastern and northern Tibetan Plateau^41^. Forty-four watersheds, mostly in North China and southeastern Tibet, had significantly decreasing trends of year-long SWA, ranging from −0.0023 ± 0.0018 km^2^ year^−^^1^ in the Suifenhe watershed in Heilongjiang Province to −2.4 ± 1.3 km^2^ year^−^^1^ in the Kashgar River watershed in Xinjiang. The decreasing trends of SWA in North China were caused by the disappearance of a number of lakes, which was driven by both natural and anthropogenic factors^18,32^. The remaining 104 watersheds had no significant trends in year-long SWA during 1989–2016. Therefore, in general, the water-rich regions of the southeastern China were becoming richer (gainers), whereas the water-poor regions of the northern China were becoming poorer (losers).

### Changes of TWS and SWA during 2002–2016

**Fig. 3:**
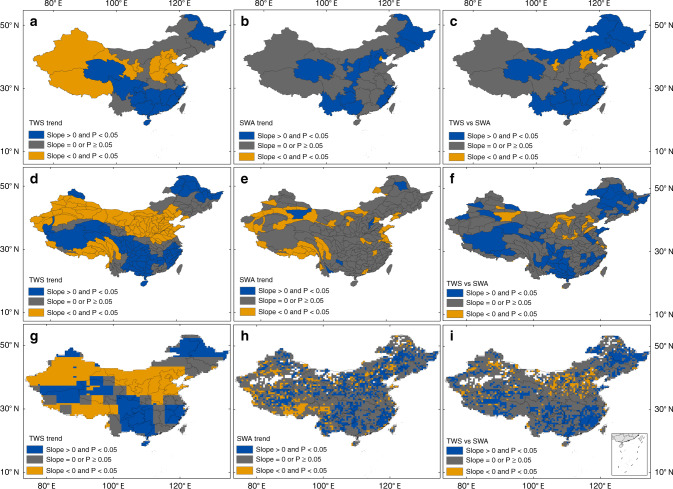
Trends of year-long surface water area and terrestrial water storage, and their linear regression trends at the provincial, watershed, and 0.5° gridcell scales during 2002–2016 with *t*-test at the 5% significance level. **a** Trend of terrestrial water storage (TWS) at the provincial scale. **b** Trend of surface water area (SWA) at the provincial scale. **c** Trend of linear regressions between SWA and TWS at the provincial scale. **d** Trend of TWS at the watershed scale. **e** Trend of SWA at the watershed scale. **f** Trend of linear regressions between SWA and TWS at the watershed scale. **g** Trend of TWS at the 0.5° gridcell scales. **h** Trend of SWA at the 0.5° gridcell scales. **i** Trend of linear regressions between SWA and TWS at the 0.5° gridcell scales.

We assessed the spatial–temporal dynamics of the year-long SWA and the terrestrial water storage (TWS) from the GRACE satellite during 2002–2016 in China. At the provincial scale, ten provinces had significantly decreasing trends of TWS, which ranged from −0.1 ± 0.08 cm year^−^^1^ in Gansu to −1.7 ± 0.7 cm year^−^^1^ in Shandong (Fig. 3a). For the provinces in North China Plain, agriculture intensification and increased groundwater use were the major driving forces for the decreased TWS^26^. For example, in Shandong Province the amount of groundwater use exceeded the amount of groundwater recharge by the natural processes over the past several decades, and excessive withdrawal of groundwater resulted in the largest decreasing trend of TWS (−1.7 ± 0.7 cm year^−^^1^) in Shandong^34,42^. The mass losses of glaciers in Tibet and Xinjiang contributed considerably to the losses of TWS^37,41^. Xinjiang is one of the world’s largest producers of coal, thus groundwater use by coal mining in the area might have also contributed to the decrease of TWS^43^. Qinghai had a significantly increasing trend of TWS (Fig. 3a), which was related to the increased SWA with high R square and small standard error (Fig. 3b, c; Supplementary Fig. 6a, b) and other factors^44^. Guangxi and Guizhou Provinces had significantly increasing trends of TWS and SWA, where substantial vegetation recoveries driven by various ecological engineering projects were also observed^45^. In comparison, Inner Mongolia, Gansu, and Shaanxi, where major ecological engineering projects were also implemented^46^, did not have significant changes of TWS and SWA during 2002–2016, which raises the concern on the effectiveness of these projects on conservation of water resources in these provinces.

At the watershed scale, the interannual trends of TWS during 2002–2016 had a distinct spatial pattern (Fig. 3d). Most of the watersheds in southern Tibet and northern China had significantly decreasing trends of TWS (Fig. 3d), but few of them had significantly increasing trends of SWA (Fig. 3e). The significantly negative correlations between TWS and SWA in these watersheds (Fig. 3f) suggest that excessive use of groundwater might have contributed to the losses of TWS in these watersheds^27^. A recent study that examined groundwater level data from 801 wells in China had reported large decreasing trends of groundwater levels among the wells in those regions^29^. Significantly positive correlations between TWS and SWA over the watersheds in Northern Tibet and Qinghai with high R squares suggest that SWA contributed significantly to the TWS dynamics (Supplementary Fig. 6c). A recent study reported strong relationship between TWS and surface water storage of large lakes (>10 km^2^) in the Tibetan Plateau^20^. Many watersheds along the Yangtze River had significantly increasing trends of TWS (Fig. 3d). The temporal dynamics of TWS and SWA in many watersheds in Guangxi and Guizhou Provinces were highly correlated (Fig. 3d–f). In total, 18 watersheds (27% of China’s total land area) had significantly positive correlations between TWS and SWA, and 59 watersheds (7% of China’s total land area) had significantly negative correlations between TWS and SWA (Fig. 3f).

We further investigated the temporal relationships between TWS and year-long SWA within individual 0.5° gridcells (longitude/latitude). TWS increased significantly in 1268 gridcells (34.7% of 3654 gridcells in China), most of which were distributed in northern Tibet, South China, and Northeast China (Fig. 3g). TWS decreased significantly in 1408 gridcells (38.5%), mostly in Xinjiang Province, southern and southeastern Tibet, and the Yellow River Basin. The southeastern Tibet and Tianshan Mountains in Xinjiang had relatively higher decrease rates than other regions because of high retreat rates of glaciers^39^. Year-long SWA increased significantly in 996 gridcells (27.3%), mostly in northwestern Tibet, and the Sichuan, Haihe, and Songhuajiang Basins (Fig. 3h). SWA decreased significantly in 409 gridcells (11.2%), mostly in southeastern Tibet, the Tian Shan and Kunlun Mountains. The substantial losses of both surface and terrestrial water resources in southeastern Tibet clearly pose threats to water security and economy in Southern China, as southeastern Tibet is the headwater of many large rivers in the region. The temporal relationships between TWS and SWA was significantly positive in 963 gridcells and negative in 268 gridcells (Fig. 3i). Overall, our results revealed the spatial–temporal divergence and consistency between TWS and SWA during 2002–2016 in China, and extensive and continued losses of TWS, together with small to moderate changes of SWA in North China, indicated long-term water stress in the region.

### Anthropogenic and climatic drivers for TWS and SWA dynamics

**Fig. 4:**
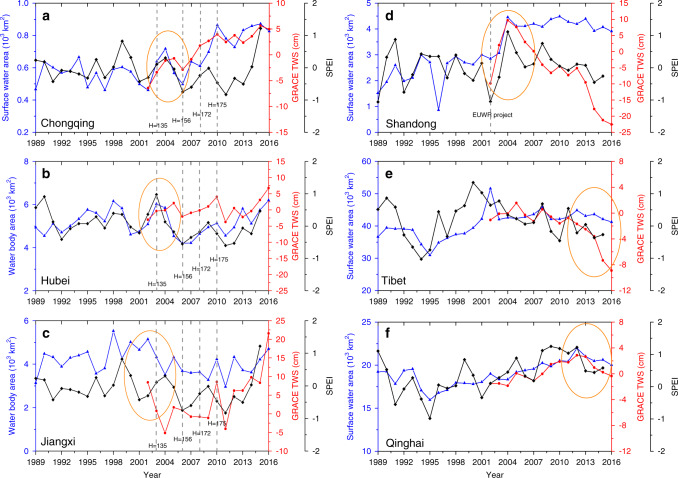
Surface water area, terrestrial water storage, and the standardized precipitation–evapotranspiration index at the provincial scale. **a** Chongqing. **b** Hubei. **c** Jiangxi. **d** Shandong. **e** Tibet. **f** Qinghai Province. The SPEI is the standardized precipitation-evapotranspiration index. The dashed lines and the numbers in subfigure (**a**–**c**) showed the water level (H) of the Three Gorge Reservoir and their corresponding years. The EUWR project in Shandong Province is the Ecological Urgent Water Replenishing (EUWR) project to divert water from the Yangtze River to Nansihu Lake during 2002–2003.

Large-scale hydrological projects (e.g., dam construction and long-distance water transfer) have been carried out in China in an effort to meet the growing needs of water resources for an increasing population^4,47,48^. One example is the Three Gorges Dam (TGD) in western Hubei Province, the largest hydroelectric dam in the world (Supplementary Fig. 7). It had large impacts on its neighboring provinces and watersheds along the Yangtze River since its first impoundment in 2003. The water level of the TGD reservoir increased to 156 m in September 2006, 172.5 m in September 2008, and 175 m (the maximum height by dam design) in October 2010^21,49,50^ (Fig. 4a–c). Chongqing, which is an upstream municipality of the TGD, had stepwise increases of TWS and SWA during 2002–2010 (Fig. 4a), corresponding well to the water level changes of the TGD reservoir. These data suggested that the TGD clearly increased upstream SWA and TWS. In Hubei Province, where TGD is located, TWS increased in 2003 but then remained relatively stable (Fig. 4b). However, SWA dropped substantially after 2003, and did not recover until the mid-2010s. Jiangxi is a downstream province of the TGD, and both TWS and SWA have dropped substantially since 2003 (Fig. 4c). These results clearly indicated that the TGD affected water resources in the upstream and downstream areas, especially SWA and TWS in the downstream of TGD^31,51^.

Other human water uses, for agriculture, industry, and human settlements, also affect the temporal dynamics of SWA and TWS. In the North China Plain, more than 60% of water resources used for human activities came from groundwater^52^. For example, in Shandong Province^42^, SWA increased slightly from early 1990s and early 2000s, and remained stable in late 2000s (Fig. 4d). Shandong suffered from a severe drought in 2002, as shown by very low SPEI value in 2002. The Ecological Urgent Water Replenishing (EUWR) project was implemented to divert water from the Yangtze River to Nansihu Lake during 2002–2003 to sustain the lake^33^, which contributed to the elevated increase of SWA and TWS during 2002–2004. After the EUWR project, SWA remained stable during 2004–2016 (Supplementary Fig. 8). However, TWS continued to decrease substantially after 2004, clearly suggesting the overexploitation of groundwater^34,42^. The production of grain, meat, fruit, and vegetables in Shandong was ~8%, 9%, 12%, and 13% of the total production in China, respectively^53^, and water use for agriculture (mostly irrigation) contributed to prolonged depletion of groundwater in the province. Therefore, although water transfer project increased SWA after 2004, excessive withdrawal of groundwater has resulted in the observed decrease of TWS in Shandong Province^52^, which poses serious challenges for decision makers and stakeholders in the region who tackle and cope with increased water stress and rising concerns on water and food security.

Climate change has affected glacial dynamics, annual precipitation, and surface water resources in the Tibetan Plateau^20,41^, which is known as “the Third Pole” of the world and “Water Tower of Asia” because of its abundant rivers, lakes, and glaciers^37^. SWA in Tibet dropped substantially during 1994–1995, following severe droughts during 1993–1995, as shown in large negative standardized precipitation evapotranspiration index (SPEI) values (Fig. 4e), which takes into account of both precipitation and potential evapotranspiration^54^. SWA in Tibet recovered in the late 1990s as SPEI became positive in those years. TWS and SWA remained relatively stable during 2002–2012 but started to drop substantially after 2012 at a rate of −1.93 cm year^−^^1^ (TWS) and −8.2 × 10^2^ km^2^ year^−^^1^ (SWA), respectively. SWA in Qinghai Province also dropped substantially in 1994–1995 and then gradually recovered in late 1990s and increased in 2000s (Fig. 4f). Both SWA and TWS peaked in 2012 and then decreased slowly in 2013–2016. Therefore, as there were no large dam or reservoir construction projects in the Tibetan Plateau^2^, precipitation was the main reason for variations in SWA and TWS in the Tibetan Plateau^20^, and glacier meltwater driven by rising temperature also contributed to the changes in SWA and TWS^20,37^.

Interannual climate variability and new reservoirs are considered as major factors contributing to the interannual variations of SWA at the provincial scale^27^. For example, in Jiangxi Province, extensive flood events in 2010 resulted in a large gain of SWA and TWS, but severe drought in 2011 resulted in a large loss of SWA and TWS (Fig. 4c). In Guangxi Province, additional new reservoirs/dams caused an elevated (stepwise) increase of SWA over years (Supplementary Note 1). Thus, here we used multi-variate stepwise regression models to identify the effects of four variables (precipitation, temperature, year-long SWA of the previous year, and areas of new reservoirs) on the changes of SWA in each province of China. These variables had little collinearity with small variance inflation factor (VIF) values (Supplementary Fig. 9). The statistical analysis indicated that annual precipitation had a significantly strong positive effect on SWA in 10 provinces (Supplementary Fig. 10a), and the increased precipitation in Heilongjiang of Northeast China had the largest contribution to the increased SWA than do other provinces^55^. Annual mean temperature had a strong negative effect on SWA in Liaoning Province (Supplementary Fig. 10b). The areas of new reservoirs in 10 provinces in China had significantly positive effects on SWA, meaning that the areas of new reservoirs significantly contributed to the increase of SWA in these 10 provinces (Supplementary Fig. 10c). In addition, year-long SWA in the previous year also had significantly positive effects in 13 provinces, especially in northern and western China (Supplementary Fig. 10d), indicating that there were strong legacy effects on the SWA dynamics^27^.

### Changes of water resources and population

**Fig. 5:**
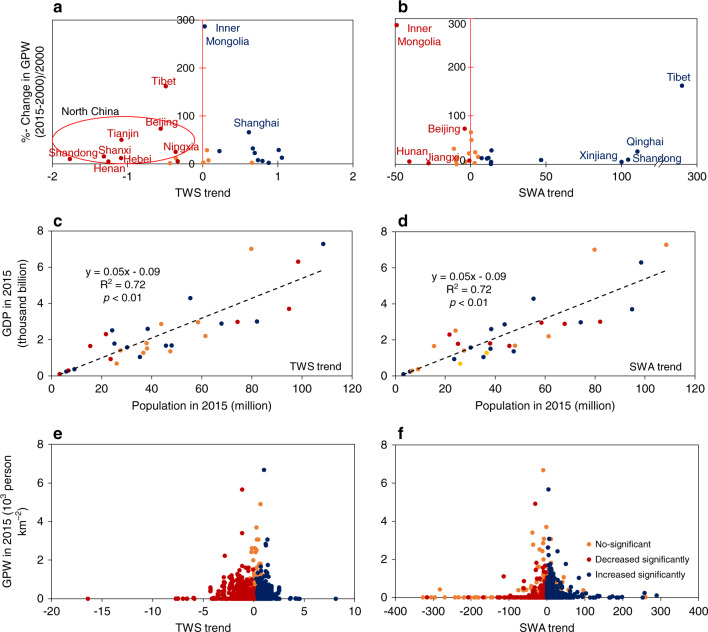
Relationships between surface water area trends, GRACE terrestrial water storage trends, gross domestic product, and the Gridded Population of the World. **a** Relationship between population density changed from 2000 to 2015 and terrestrial water storage (TWS) trends at the provincial scale. **b** Relationship between population density changed from 2000 to 2015 and surface water area (SWA) trends at the provincial scale. **c** Relationship between population, gross domestic product (GDP) in 2015, and TWS trends at the provincial scale. **d** Relationship between population, GDP in 2015, and SWA trends at the provincial scale. **e** Relationship between TWS trends and Gridded Population of the World (GPW) in 2015 at the 0.5° gridcell scale. **f** Relationship between SWA trends and GPW in 2015 at the 0.5° gridcell scale.

We investigated the relationships between the change in water resources (TWS, SWA) during 1989–2016 and the change in population during 2000–2015 at the provincial scale (Fig. 5a, b). In the 9 provinces with significantly decreasing trends in TWS (Fig. 5a), there was a total increase of 53.4 million people. In the 14 provinces with significantly increasing trends in TWS (Fig. 5a), there was a total increase of 146.3 million people. In the 7 provinces with significantly decreasing trends in SWA (Fig. 5b), population increased by 92.2 million. In the 14 provinces with significantly increasing trends of SWA (Fig. 5b), population increased by 57.1 million. In Beijing, population increased by 10 million and both TWS and SWA decreased substantially during 1989–2016. In total, over 135 million population lived in 15 provinces that experienced significant losses of TWS or SWA during 1989–2016.

To further investigate the likely challenge China’s population and economy may face in terms of water resources, we analyzed population and gross domestic product (GDP) in 2015 and the changes of TWS and SWA during 1989–2016. Human population and gross domestic product (GDP) data in 2015 had a strong linear relationship at the provincial scale (Fig. 5c, d). However, the temporal changes of water resources as measured by the trends of TWS and SWA varied among the provinces (Fig. 5c, d), which indicates that each province experienced different water resource challenges for its population and economy. Guangdong Province had the largest population, the highest GDP, and a significantly increasing TWS trend and a non-significant change in SWA (Fig. 5c), which suggests that water resources are unlikely to be a major constraint for Guangdong. On the contrary, Shandong Province ranked second in population and third in GDP, but it had a significantly decreasing trend in TWS (Fig. 5c) because of overexploitation of groundwater^34,42^, which suggests that Shandong Province is likely to face major challenge for its water security and economic development. Henan Province ranked third in population and 5th in GDP, and it also had a significantly decreasing trend in TWS (Fig. 5c), which suggests that Henan Province is also likely to face major challenges for its water security and economic development. Both Shandong and Henan Provinces had significantly increasing trends in SWA, but the significantly decreasing trends in TWS in these two provinces suggest that they need to have large structural changes in agriculture, which uses a lot of groundwater for irrigation and industry. Geographically, at the 0.5° gridcell scale, approximately 460 million (34.9%) people live in 1408 (38.5%) gridcells with significantly decreasing trends in TWS (Fig. 5e), and 109 million (8.3%) people live in 409 (11.2%) gridcells with significantly decreasing trends in SWA in 2015 (Fig. 5f; Supplementary Table 1).

Surface water resources and water security have been a major concern in China over the past decades^18,29,35,36,38^. To date, our surface water data set at 30-m spatial resolution during 1989–2016 for China is an accurate, updated, reliable, and spatially detailed data set. Our estimate of ~0.155 × 10^6^ km^2^ year-long SWA in China in 2016 reveals that surface water resources in China are very limited, where over 1.4 billion people now live. In comparison, a recent study that used Landsat images and the same mapping algorithms reported ~0.257 × 10^6^ km^2^ year-long surface water area in the contiguous United States^27^, where ~330 million people live. Our results also reveal the large and geographically divergent trends in terrestrial water storage in China during 2002–2016. Large and continued losses of SWA and TWS and decoupling (inconsistency) of temporal dynamics between TWS and SWA indicate decade-to-century-scale deficit of groundwater resources in the northern parts of China. As of 2015, ~569 million people lived in the areas that experienced significant losses of TWS or SWA during 1989–2016. A number of climate and hydrological models have predicted large interannual variations in climate in northern China, including frequent droughts and heatwaves^35,45^. Further population growth and climate changes pose enormous challenges for water resources management in China. The surface water data set and the findings from this study can be used to assist water resources managers, stakeholders, decision makers, and the public to develop evidence-based planning and management of limited water resources in China, in particular under increasing water demand and use, more frequent droughts and heatwaves, and climate change.

## Methods

### Study area

This study includes 23 provinces, 5 autonomous regions, 4 municipalities, and the special administrative regions of Hong Kong and Macau in China (Supplementary Fig. 11a). It has a large variation in topography, ranging from the high-elevation Tibetan Plateau to the immense plains and seashore (Supplementary Fig. 11b). Climate in China also varies substantially, ranging from tropical climate in the far south to subarctic in the far north and alpine in the higher elevations of the Tibetan Plateau. The spatial patterns of annual precipitation and mean temperature are complex (Supplementary Fig. 11c, d).

### Landsat data

This study acquired all the available Landsat 5/7/8 Surface Reflectance (SR) images between January 1, 1984 and December 31, 2016 in China (∼338,000 images) in the Google Earth Engine (GEE) cloud processing platform. For each image, the quality assurance (QA) band was used to identify and remove the bad-quality observations, including clouds, cloud shadows, cirrus, snow/ice, and scan-line corrector (SLC)-off gaps^56^. Shuttle Radar Topography Mission (SRTM) digital elevation model (DEM), as well as the solar azimuth and zenith angles of each image, were used to remove terrain shadows^27^. There were large spatial variations in the number of good-quality observations among individual pixels over the study period. The larger numbers of good-quality observations in North China were due to the overlapping Landsat images at the high latitudes and fewer clouds (Supplementary Fig. 12a). The numbers of Landsat 5, 7, and 8 images for each year were highly variable over the study period, as more Landsat images were acquired after the launch of Landsat 7 in 1999 and Landsat 8 in 2013 (Supplementary Fig. 12b). Pixels with no good observation accounted for about 90.1% of all pixels during 1984–1985 (Supplementary Fig. 12c), and they accounted for 40.6% during 1986–1988, but they accounted for only 3.6% of all pixels during 1989–2016. Because of large number of pixels with the limited observations during 1984–1988, we excluded these years when we carried out the interannual trend analysis at the provincial and watershed scales (1989–2016). Supplementary Figure 13 showed the frequency of the numbers of good-quality observation in each season of China from 1989 to 2016, and about 90% of all pixels had at least 1 good-quality observations in each season, and 95% of all pixels had at least 1 good-quality observations in each season after 1990 except 2012 when only Landsat 7 was available (Supplementary Fig. 12b), so the quality of Landsat images in this study could be used to detect both seasonal and year-long surface water bodies.

In this study, we used three widely used indices (NDVI, EVI, mNDWI) to identify surface water bodies. These indices are defined as:

1 $${\mathrm{NDVI}} = \frac{{\rho _{{\mathrm{nir}}} - \rho _{{\mathrm{red}}}}}{{\rho _{{\mathrm{nir}}} + \rho _{{\mathrm{red}}}}},$$

2 $${\mathrm{EVI}} = 2.5 \times \frac{{\rho _{{\mathrm{nir}}} - \rho _{{\mathrm{red}}}}}{{\rho _{{\mathrm{nir}}} + 6 \times \rho _{{\mathrm{red}}} - 7.5 \times \rho _{{\mathrm{blue}}} + 1}},$$

3 $${\mathrm{mNDWI}} = \frac{{\rho _{{\mathrm{green}}} - \rho _{{\mathrm{swir}}}}}{{\rho _{{\mathrm{green}}} + \rho _{{\mathrm{swir}}}}},$$

where *ρ*_blue_, *ρ*_green_, *ρ*_red_, and *ρ*_swir_ are blue, green, red, near-infrared, and shortwave infrared bands of Landsat images.

### Terrestrial water storage (TWS) data

The Gravity Recovery and Climate Experiment (GRACE) Tellus Monthly Mass Grids provide monthly gravitational anomalies relative to a 2004–2010 time-mean baseline and the monthly liquid water equivalent thickness (LWET) data in this data set are units of “Equivalent Water Thickness”, which represent the deviations of mass in terms of vertical extent of water in centimeters^57^. The 0.5° monthly LWET data during 2002–2016 (https://grace.jpl.nasa.gov/) were used to calculated annual average LWET data (terrestrial water storage, TWS), which were used to explore its relationships with surface water area.

### Climate data

The National Centers for Environmental Prediction-Department of Energy (NCEP/DOE) Atmospheric Model Inter-comparison Project (AMIP-II) Reanalysis (R-2)^58^, from their website at www.esrl.noaa.gov/psd/, were used to calculate annual average precipitation and temperature during 1989–2016 (Supplementary Fig. 11c, d), which were used in this study as predictor variables for interannual variations of surface water area in China.

The Standardized Precipitation Evapotranspiration Index (SPEI) was designed to consider both precipitation and potential evapotranspiration and can capture the main impact of increased temperatures on water demand^59^. The global 0.5° gridded SPEI data set, calculated using CRU TS 3.23 data set, covers the period 1901−2015 and downloaded at http://sac.csic.es/spei/. SPEI data set in China during 1989–2015 was used in this study to investigate the effect of climate change on surface water area and terrestrial water storage in China at the provincial scale.

### Reservoir data

Reservoir data in China was acquired from the Global Reservoir and Dam Database (GRanD) v1.3^60^ (http://globaldamwatch.org/grand/). We used the areas of new reservoirs from the GRanD data set (Supplementary Fig. 14) to explore the contribution of new reservoirs to the changes of SWA at the provincial scale during 1989–2016.

### Population and gross domestic product (GDP) data

Population density data were acquired from the fourth version Gridded Population of the World (GPWv4) collection (http://sedac.ciesin.columbia.edu/data/collection/gpw-v4). We used the GPW with 0.5-degree spatial resolution in this study to explore its relationship with the changes of GRACE TWS trends and surface water areas trends. The gross domestic product (GDP) data of 2015 in each province (except Taiwan Province) in China were collected from the China Statistical Yearbooks from the National Bureau of Statistics of the People’s Republic of China (available at: http://www.stats.gov.cn/tjsj/ndsj).

### Watershed boundary data

The watershed boundary map of China was acquired from the Resource and Environment Data Cloud Platform of the Institute of Geographic Sciences and Natural Resources Research, Chinese Academy of Sciences (http://www.resdc.cn/data.aspx?DATAID=278). It contains 209 third-order watersheds in China, and it was used to analyze spatial–temporal dynamics of surface water area and terrestrial water storage at the watershed scale.

### Algorithm to generate maps of surface water body

The water index (mNDWI) has been widely used to detect surface water bodies^61^, but it has commission errors (user’s accuracy) in pixels having a mix of surface water bodies and other land cover types, especially when the pixel comprises vegetation and water body^62^. To reduce the effects of vegetation on identifying surface water body, mNDWI was combined with two greenness-based vegetation indices (EVI and NDVI) to detect surface water bodies. For spectral signature analysis of land cover types, we selected training samples in each of 574 path/row (tiles) of the Landsat Worldwide Reference System (WRS-2), which cover the entire China. Three water points and two non-water points were selected visually within each tile based on the very high spatial resolution images of 2016 in the Google Earth. Altogether, we selected 1722 random water points and 1148 random non-water points to study the distribution of spectral indices (Supplementary Fig. 15). Approximately 99.42% of the water points had mNDWI > EVI, 98.22% of the non-water pixels had mNDWI < EVI, 99.07% of the water pixels had mNDWI > NDVI, and 99.09% of the non-water pixels had mNDWI < NDVI. Thus, we determined that mNDWI > EVI and mNDWI > NDVI were good criteria to distinguish water body from non-water points. As 97.76% of the water pixels had EVI < 0.1 (Supplementary Fig. 15b), EVI < 0.1 can be used to exclude pixels mixed with water and vegetation. The final open surface water body detection algorithm was ((mNDWI > EVI or mNDWI > NDVI) and EVI < 0.1). This mNDWI-VIs algorithm was first implemented to map the surface water body in the state of Oklahoma, US^23^, and was then used in the contiguous United States with high accuracy^27^. Furthermore, this mNDWI-VIs algorithm had been compared with other water mapping algorithms (e.g. NDWI, mNDWI, TCW, and AWEI) using Landsat and Sentinel-2 images, and the results showed that this algorithm can identify open surface water bodies in Landsat images with high producer’s accuracy (98.1%) and user’s accuracy (91.0%)^63^.

Surface water body in each observation of individual pixels was first determined using ((mNDWI > EVI or mNDWI > NDVI) and EVI < 0.1) (Eq. (4)), then the surface water frequency of each pixel, which is defined as the proportion of water observations, was calculated using Eq. (5). Finally, we generated a surface water frequency map of all pixels in China for each year from 1989 to 2016 in the GEE platform.

4 $${\mathrm{Water = }}\, \left( {{\mathrm{mNDWI}} \, > \, {\mathrm{EVI}}\;{\mathrm{or}}\;{\mathrm{mNDWI}} \, > \, {\mathrm{NDVI}}} \right)\;{\mathrm{and}}\;{\mathrm{EVI}} \, < \, {\mathrm{0}}{\mathrm{.1}},$$

5 $${\mathrm{FW}} = \frac{{{{N}}_{{\mathrm{water}}}}}{{{\it{N}}_{{\mathrm{good}}}}},$$

where FW is surface water frequency scaled from 0 to 1.0 at individual pixels with good-quality observations, *N*_water_ is the number of observations identified as surface water body (Eq. (4)) in a year, *N*_good_ is the number of good-quality observations in a year, respectively.

### Accuracy assessment of surface water body maps

Stratified random sampling approach, along with very high spatial resolution (VHSR) images from Google Earth, were most widely used and robust approach in accuracy assessment of land cover classification, such as the global surface water data set^2^, the global tidal flats data set^64^, and other maps at national and regional scales^27,65,66^. In this study, stratified random sampling approach was used to validate the year-long and seasonal surface water body maps, respectively, following the strategy used in the JRC data set^2^. A grid (0.2 latitude by 0.2 longitude) was generated in China to collect samples for accuracy assessment. In order to determine the user’s accuracy (measure of commission error) of the resultant maps in this study, for each 0.2 by 0.2 gridcell, one point was generated randomly within the surface water body map in 2016 acquired using the above-mentioned mNDWI-VIs algorithm, and total 8197 random points were selected finally. Each point was checked and interpreted visually in Google Earth as water and non-water land cover types. Similarly, the same grid (0.2 latitude by 0.2 longitude) was used again to determine the producers’ accuracy (measure of omission error). A random point was selected within the existing published global surface water map^67^ in each gridcell, following the JRC data set. Finally, 10,200 random points were generated in this study. Each point was checked with the surface water map acquired using mNDWI-VIs algorithm and all the points confirmed as surface water body were used to estimate the producer’s accuracy. All the validation points were broken down by water class (year-long and seasonal water body) (Supplementary Tables 2 and 3). Supplementary Figure 16 shows the geographic distribution of all sample points.

The user’s accuracy (measure of commission error) for year-long and seasonal surface water body in this study was 99.71% (±0.12) and 98.57% (±0.47), respectively. The producer’s accuracy (measure of omission error) for year-long and seasonal surface water body was 99.12% ± 0.43 and 86.43% ± 3.57, respectively (Supplementary Table 4). The user’s and producer’s accuracies for year-long surface water body were larger than those for seasonal surface water body because seasonal surface water body comprised many kinds of temporary water, which may be mapped at one date, but may be missed at another^2^. In order to compare our accuracy to the JRC data set, we calculated the producer’s and user’s accuracy of the JRC data set by water seasonality class without sensors differentiating (Supplementary Table 4). The user’s accuracy and producer’s accuracy for year-long (permanent) surface water body in this study were similar with those of the JRC data set, which is well understood and expected as most of year-long (permanent) surface water body are large size and should be easy to be identified and mapped. For the seasonal surface water body, the producer’s accuracy was similar between our data set and the JRC data set, but the producer’s accuracy from our data set (86.43% ± 3.57) was much larger than that of the JRC data set (68.4%). Therefore, the surface water body data set from this study is clearly an improvement over the JRC data set in terms of year-long (permanent) and seasonal surface water body layers.

### Cross-comparison with other data sets

In addition to the stratified random sampling approach, we acquired the JRC data set^2^, the Global River Widths from Landsat data set (GRWL)^68^, the surface water layer from the Global Land Cover Facility (GLCF)^10^, and the Moderate Resolution Imaging Spectroradiometer (MODIS) 250 m land–water mask (MOD44W) data set^69^ for cross-comparison at the provincial scale (Supplementary Fig. 5). The SWA at the provincial scale in China from 1989 to 2016 had good consistency between this study and the JRC data set (*R*^2^ = 0.99, standard error = 0.56), except for the years of 1997 and 1998 when the JRC data set missed the effect of extensive floods in South China during 1997–1998. The GLCF and MOD44W data sets had smaller SWA than this study and the JRC data set because those two data sets in 2000 were generated through image mosaic and existing surface water body data sets, and SWA might be underestimated because some surface water bodies may be missed in the images at one date but be found in another^2^. The GRWL data set focused on characterizing the global coverage of large rivers and streams, so a large number of surface water bodies (e.g. small stream, lakes) were missed in the GRWL data set (Supplementary Fig. 17c, f), which resulted in the much smaller SWA estimates than those of the JRC data set and this study.

At the watershed scale, year-long SWA estimates in our data set were on the average 0.20% ± 0.85% greater than those in the JRC data set (Supplementary Fig. 18a). The difference in most watersheds were between −1 and 1%, and a few watersheds had larger differences (>1%), which were distributed in the regions with high altitudes, such as the Hailaer Watershed in Northeast China, the Kaikong River Watershed in the Tianshan Mountains, and some regions in eastern Tibet. SWA estimates in most watersheds in the GRWL data set were much smaller than those in this study (−22.41% ± 75.08), and some regions in North China even had differences < −100% (Supplementary Fig. 18b), which could be attributed to the fact that the GRWL data set missed most of small rivers and lakes (Supplementary Fig. 17c, f).

### Uncertainties of the annual surface water body maps

Our study, together with previous works^2,10,20,23,27,32^, greatly contributed to the current water resource study. However, we must also recognize that the data quality and the amount of Landsat images remain to be a concern. As Landsat has a 16-day revisit cycle, it is inevitable to miss some short-duration surface water events, such as fresh floods, when observations do not coincide with these surface water events^2^. Some water bodies are smaller than 30 m by 30 m, and thus could not be identified and mapped. For this reason, the areas of seasonal and ephemeral water bodies are likely to be underestimated in this study. The quality assurance (QA) band is an important indicator of the Landsat imagery quality and may affect mapping algorithms. Some bad-quality observations (e.g. clouds, cloud shadows) may remain after quality filtering, which might have generated some low-frequency inundation noises over the land surface. Therefore, the spatial–temporal dynamics of year-long surface water bodies in this study can provide much more reliable information than those of seasonal surface water bodies. It should also be noted that in addition to the additional historical Landsat data that will be added into GEE platform by the United States Geological Survey (USGS) Landsat Global Archive Consolidation^70^, more images from other high spatial resolution sensors (e.g., Sentinel-1, Sentinel-2) are likely to further improve remote sensing of surface water bodies in the future, which will provide more detailed geospatial data products of surface water bodies for hydrology and water security in China.

### Statistical analyses

The land area (km^2^) of each province and watershed was generated using the Projected Coordinate System Krasovsky_1940_Albers. SWA in each province and watershed from 1989 to 2016 was calculated by using the annual surface water body frequency maps. The SWA per unit land and the trend of year-long SWA at the provincial and watershed scales were analyzed using linear regression models with a *t*-test at the 5% significance level. The summed areas of year-long surface water body within 0.5° gridcells and GRACE TWS were used to analyze their relationship using linear regression models.

Interannual dynamics (variations and trends) of SWA and TWS are affected by new dams/reservoirs, water use, and climate (interannual variations (e.g., floods and droughts) and trends). Lehner et al.^60^ reported a comprehensive Global Reservoir and Dam Database (GRanD) data set, which provides the detailed information about the global reservoirs and associated dams, including geolocations (latitude and longitude) of dams and the areas of the reservoirs^60^. Its latest version (v1.3) was recently released to the public and used in this study. It contains 923 dams in China, but 51–55% dams have in-accurate geolocation information (Supplementary Note 2). In China, 782 dams in the GRanD data set had exact building years, among which only 218 (24%) dams were built after 1989 (Supplementary Fig. 19). We calculated the areas of all reservoirs in each province from 1989 to 2016, and did the multi-variate regression between SWA and climate factors (precipitation (mm) and average temperature (°C)) and other variables (year-long SWA of the previous year (10^3^ km^2^) and areas of reservoirs (km^2^)) (Eq. (6)). The positive correlations in these models were defined as slope > 0, and the negative correlation was defined as slope < 0. The uncertainties in this study were expressed as the 95% confidence interval. All the statistical analyses were carried out using R package.

6 $${\mathrm{SWA}} = {\mathrm{aX}}1 + {\mathrm{bX}}2 + {\mathrm{cX}}3 + {\mathrm{dX}}4,$$

where SWA is the year-long surface water body area (10^3^ km^2^); X1 is the average precipitation (mm); X2 is the average temperature (°C); X3 is the areas of reservoirs (km^2^); and X4 is the year-long SWA of the previous year (10^3^ km^2^).

## Supplementary information

Supplementary Information

## Data Availability

All data sets used in this study are available upon request and come from either several public data sources or as provided by original data producers (authors).

## Code availability

Code used in calculations of surface water bodies is available upon request.
