# Supplementary material for: Gainers and losers of surface and terrestrial water resources in China during 1989–2016
Source: Nat Commun. 2020 Jul 10;11:3471. doi: 10.1038/s41467-020-17103-w (PMC7351719; doi:10.1038/s41467-020-17103-w)
Supplement: Supplementary file 1 — Supplementary Information [file 41467_2020_17103_MOESM1_ESM.pdf]

SUPPLEMENTARY INFORMATION

Gainers and losers of surface and terrestrial water resources in China during 1989-2016

By Wang et al.

Includes:

Supplementary Figures 1-22

Supplementary Table 1-4

Supplementary Notes 1-2

Supplementary References

Supplementary Figure. 1

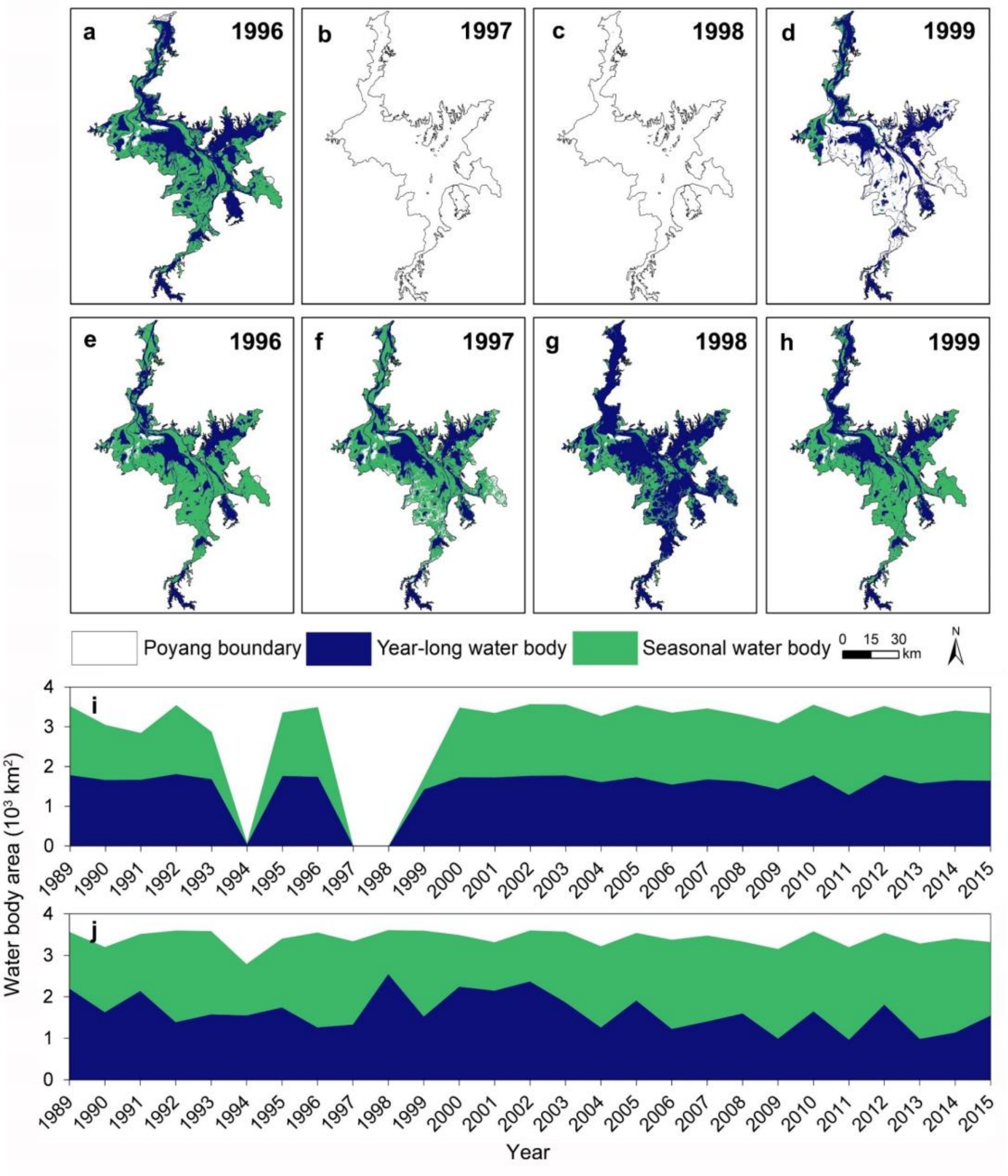

Supplementary Fig. 1 | Year-long and seasonal water body areas of Poyang Lake in the Yangtze River Basin of China. a-d, Spatial distribution of surface water bodies from the Joint Research Centre (JRC) dataset during 1996-1999. **e-h**, Spatial distribution of surface water bodies from this study during 1996-1999. **i**, Annual surface water areas from the JRC dataset during 1989-2015. **j**, Annual surface water areas from this study during 1989-2015.

Supplementary Figure. 2

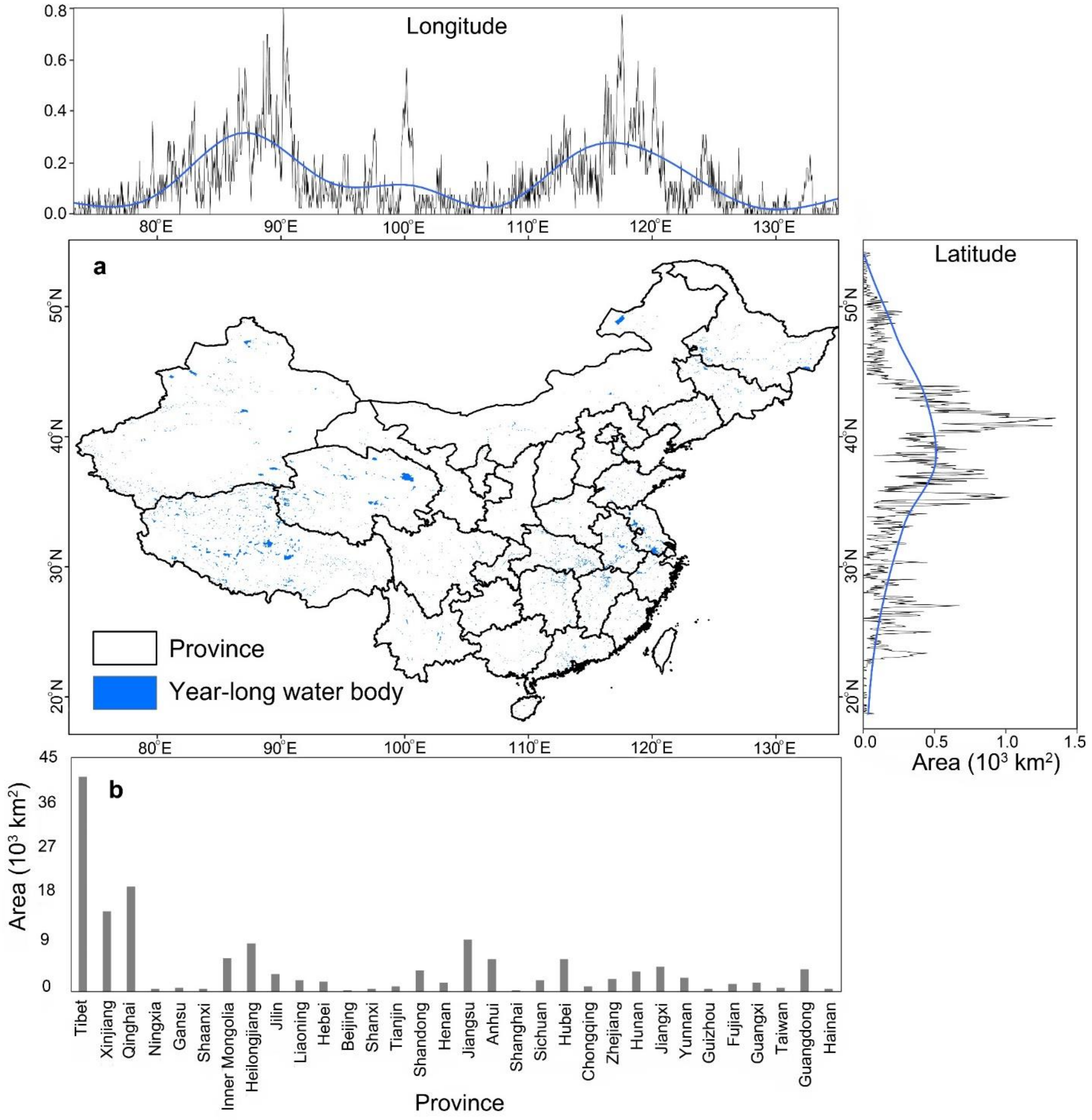

Supplementary Fig. 2 | Spatial distribution and areas of year-long surface water bodies in 2016 in China. a, Spatial distribution and summary of year-long surface water area along latitude and longitude gradients. **b**, Year-long surface water area by province. Guangdong Province includes the special administrative regions of Hong Kong and Macao.

Supplementary Figure. 3

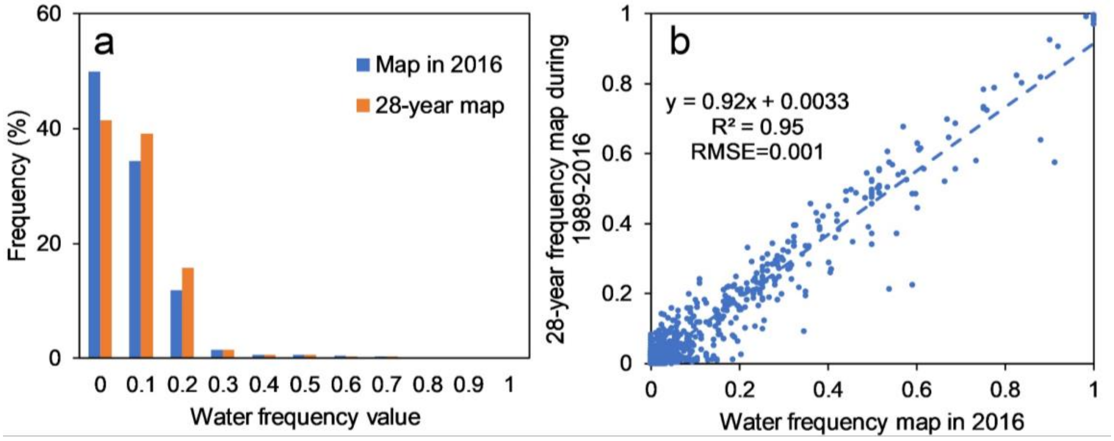

Supplementary Fig. 3 | Comparison between the water frequency map in 2016 and the 28-year water frequency map during 1989-2016. a, Histograms of surface water frequency values from the surface water frequency map in 2016 and the 28-year map (1989-2016). **b**, Relationship between the surface frequency values in 2016 and the 28-year map (1989-2016).

Supplementary Figure. 4

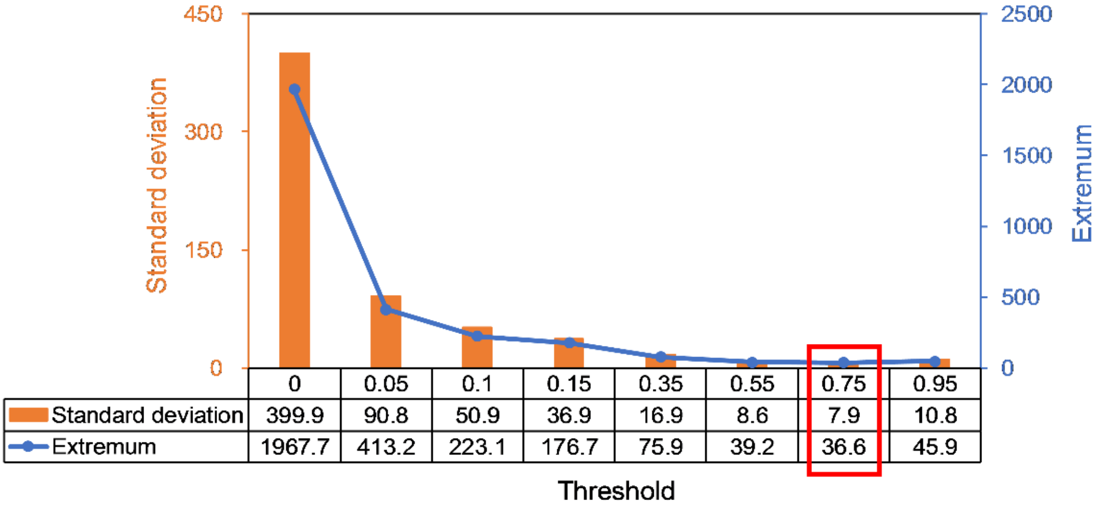

	0	0.05	0.1	0.15	0.35	0.55	0.75	0.95
Standard deviation	399.9	90.8	50.9	36.9	16.9	8.6	7.9	10.8
Extremum	1967.7	413.2	223.1	176.7	75.9	39.2	36.6	45.9

Supplementary Fig. 4 | Standard deviation and the extremum of surface water areas with different surface water body frequency thresholds from 1989 to 2016 at the national scale.

Supplementary Figure. 5

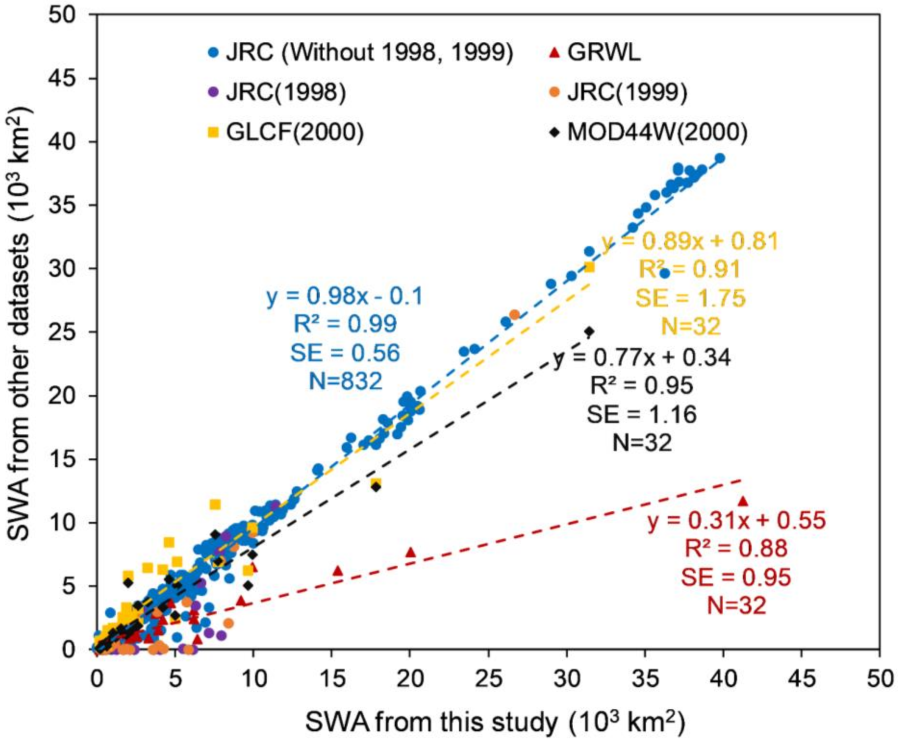

Supplementary Fig. 5 | Comparison of surface water area between this study and other datasets. Surface water area (SWA) from the JRC dataset in 1998 and 1999 were shown separately. The Global River Widths from Landsat dataset (GRWL) dataset (2016), the surface water layer from the Global Land Cover Facility (GLCF) dataset (2000), and the Moderate Resolution Imaging Spectroradiometer (MODIS) 250 m land–water mask (MOD44W) dataset (2000) were also compared with this study. SE in the figure means standard error.

Supplementary Figure. 6

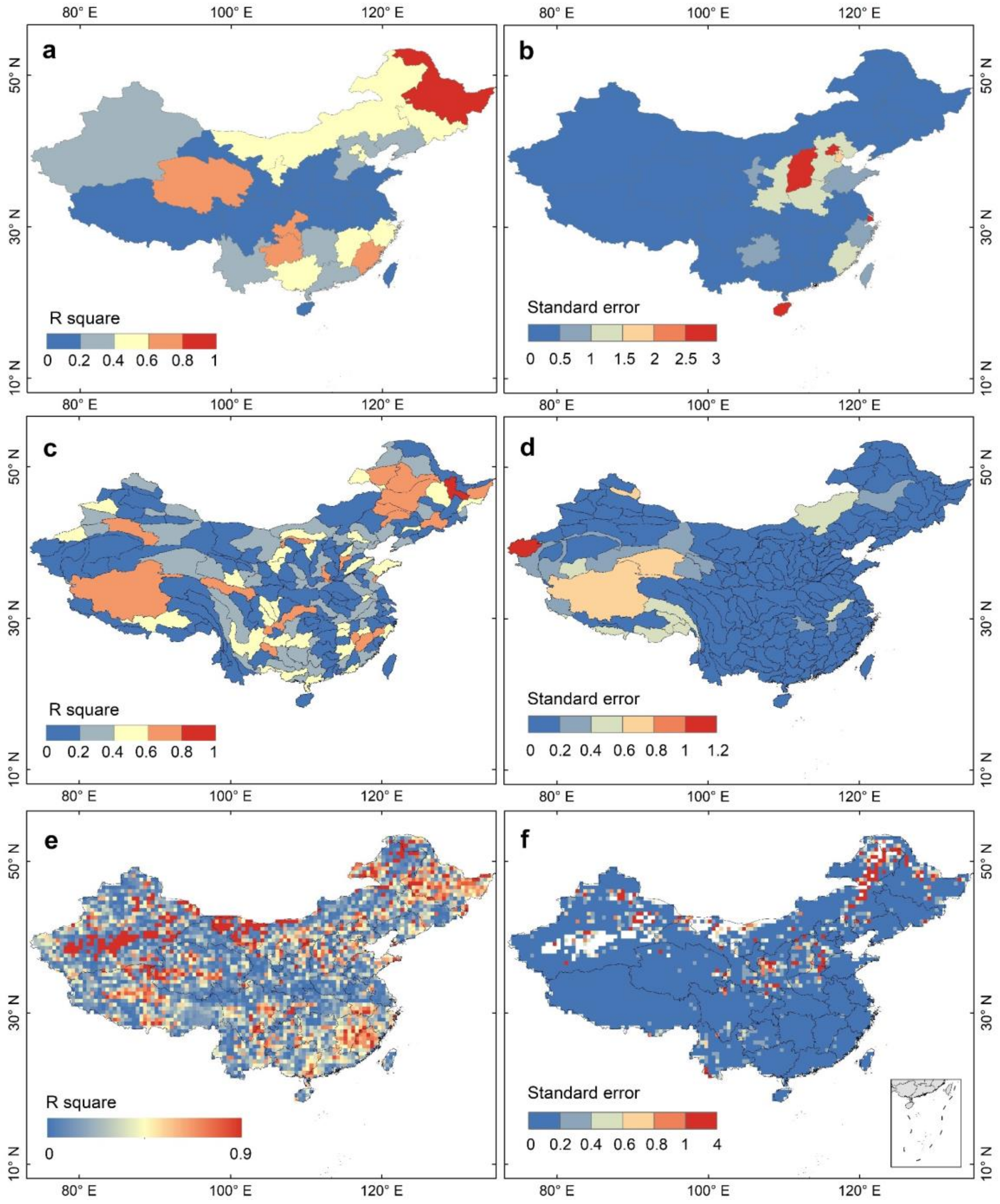

Supplementary Fig. 6 | R square and standard error of linear regression trends between surface water area (SWA) and terrestrial water storage (TWS) at the provincial (a-b), watershed (c-d), and the 0.5-degree grid cell (e-f) scales.

Supplementary Figure. 7

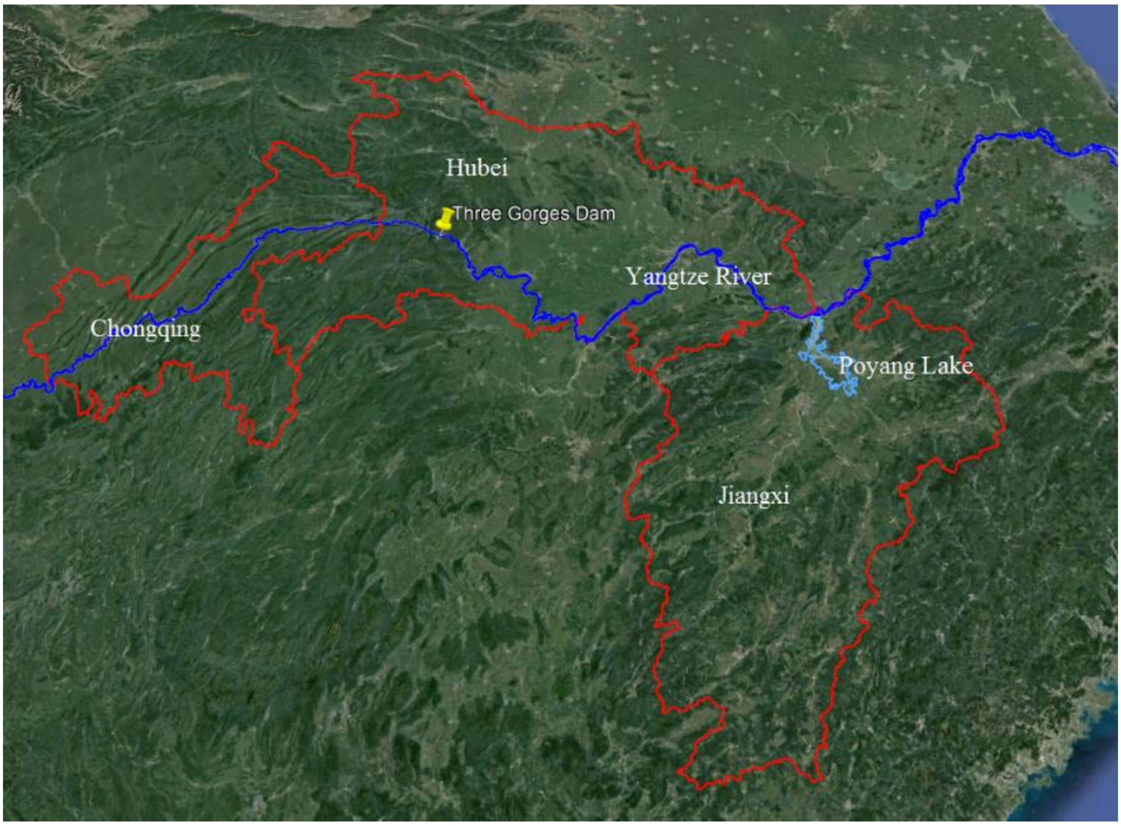

Supplementary Fig. 7 | Location of Chongqing, Hubei, Jiangxi Provinces, and Three Gorges Dam, the Yangtze River, and Poyang Lake in South China.

Supplementary Figure. 8

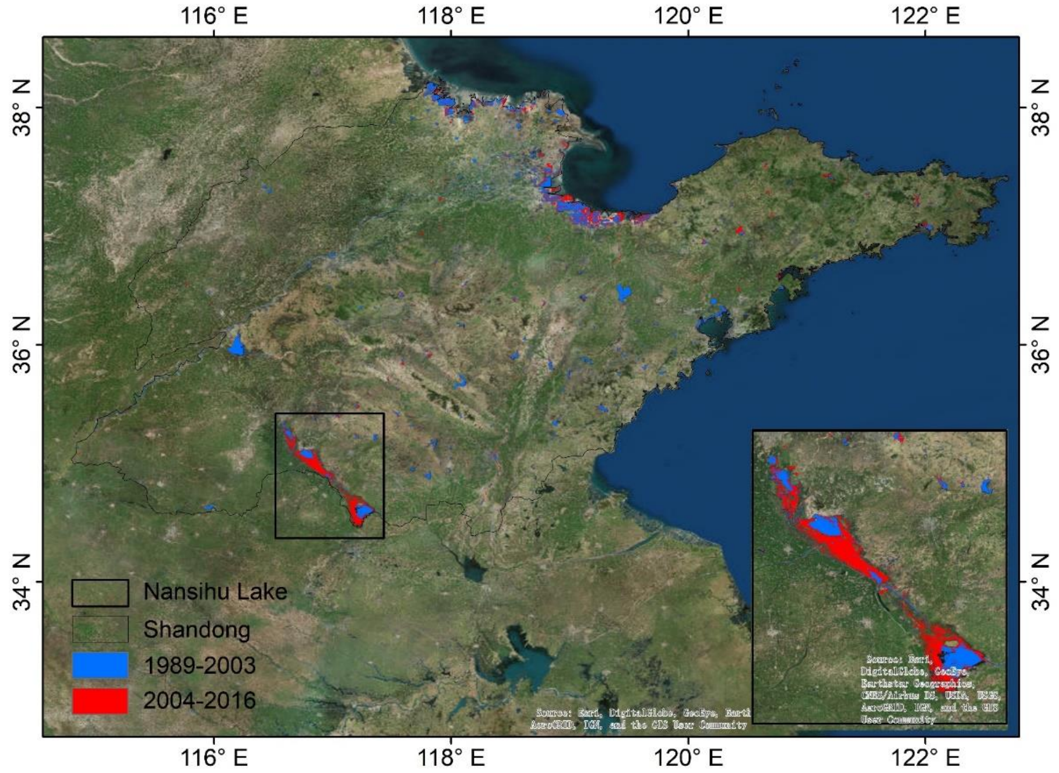

Supplementary Fig. 8 | Spatial distribution of year-long surface water bodies in Shandong Province based on the average surface frequency maps during 1989-2003 and during 2004-2016. The zoom-in shows the detail of Nansihu Lake.

Supplementary Figure. 9

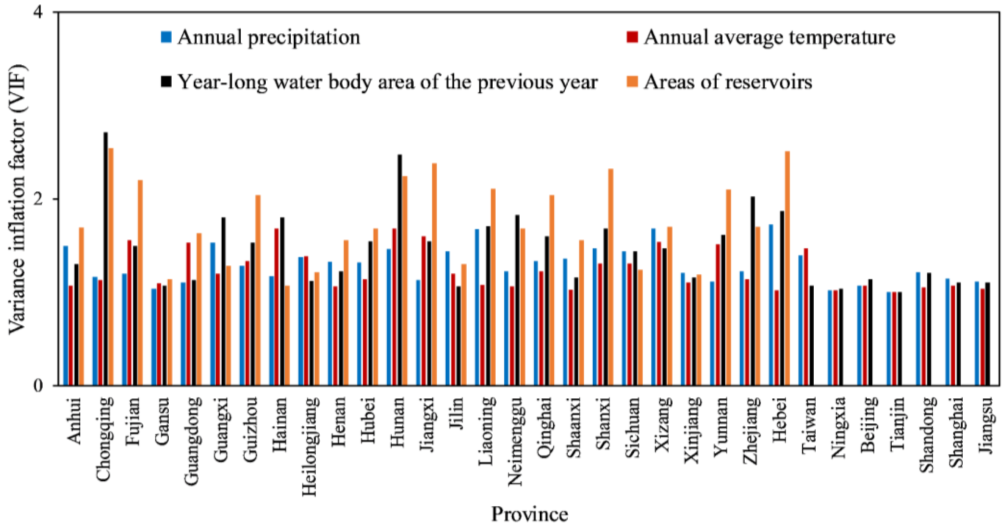

Supplementary Fig. 9 | Variance inflation factor (VIF) of multiple stepwise regression models between surface water area (SWA), climate factors (precipitation (mm) and average temperature (℃)), and other variables (year-long SWA of the previous year (10^3 km^2) and areas of new reservoirs (km^2)).

Supplementary Figure. 10

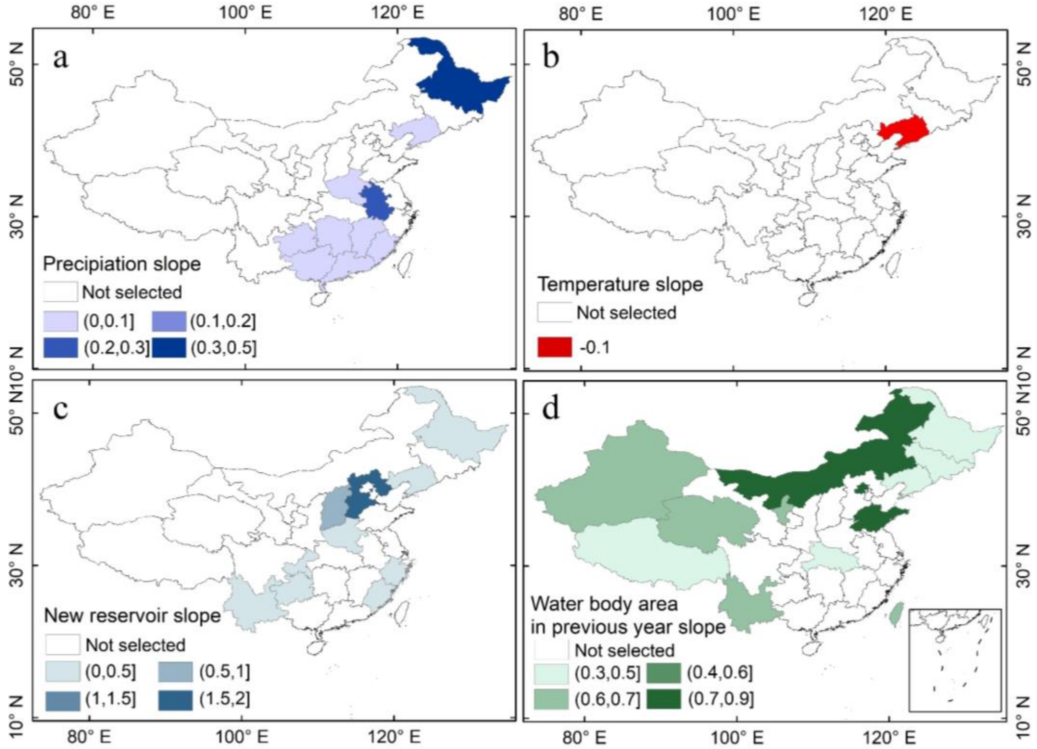

Supplementary Fig. 10 | Multiple stepwise regression models between surface water area and climate and other variables at the provincial scale. The regression model is surface water area (SWA) = aX1 + bX2 + cX3 + dX4, X1 is annual precipitation, X2 is annual average temperature, X3 is the areas of new reservoirs, and X4 is SWA in the previous year. **a**, Slope of annual precipitation with p-value < 0.05. **b**, Slope of annual average temperature with p-value < 0.05. **c**, Slope of new reservoirs areas with p-value < 0.05. **d**, Slope of year-long water body area in the previous year with p-value < 0.05.

Supplementary Figure. 11

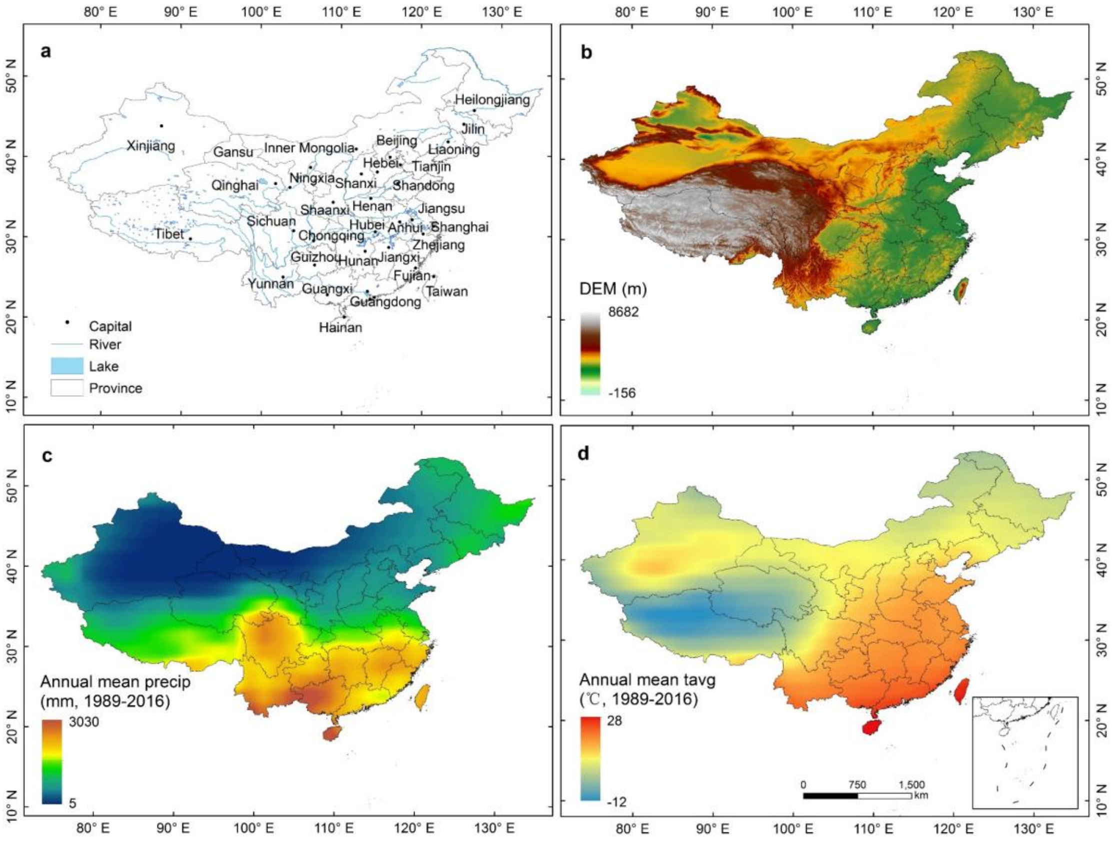

Supplementary Fig. 11 | Study area. a, Map of administration units, capitals, rivers, and lakes in China. **b**, Digital elevation model (DEM) of China. **c**, Mean annual precipitation (precip) during 1989–2016. **d**, Mean annual air temperature (tavg) during 1989–2016.

Supplementary Figure. 12

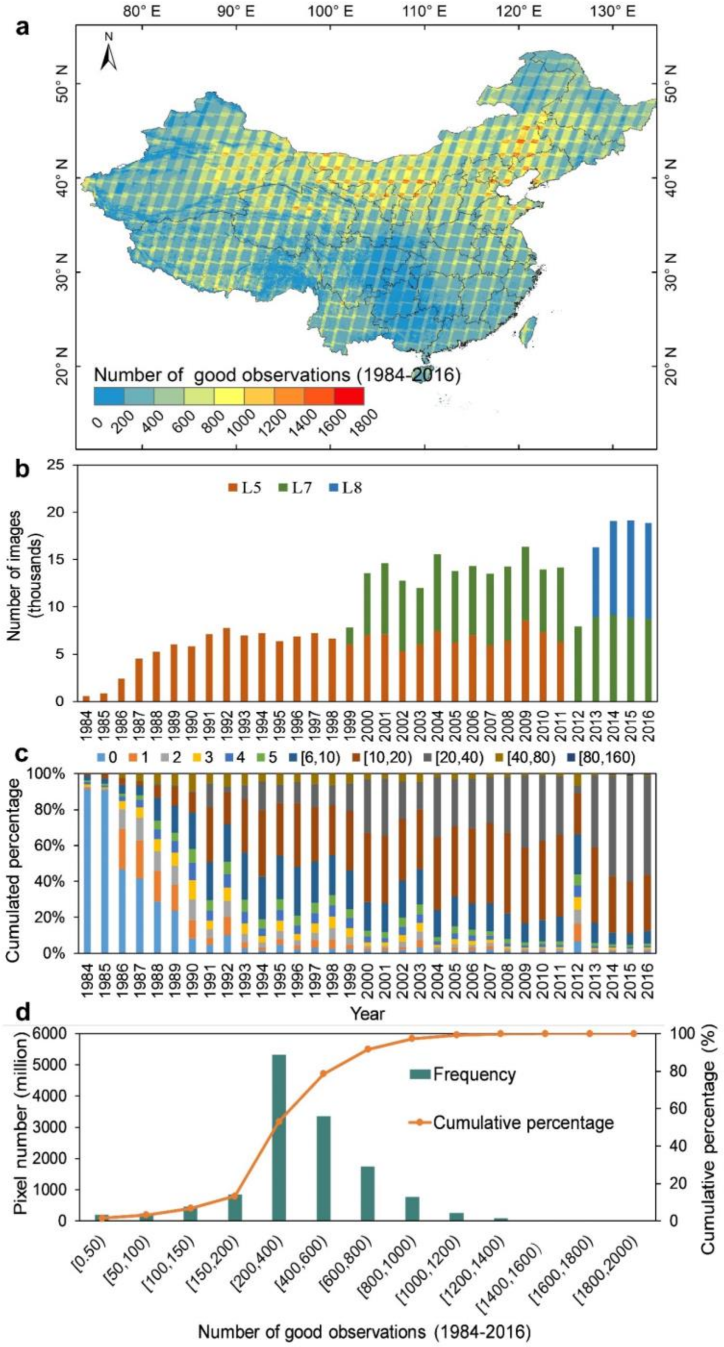

Supplementary Fig. 12 | Landsat data in China during 1984-2016. a, Spatial distribution of number of good observations. **b**, Number of Landsat 5, 7, and 8 images in each year. **c**, Distribution of good observations per pixel in each year. **d**, Distribution of good observations per pixel in 33 years.

Supplementary Figure. 13

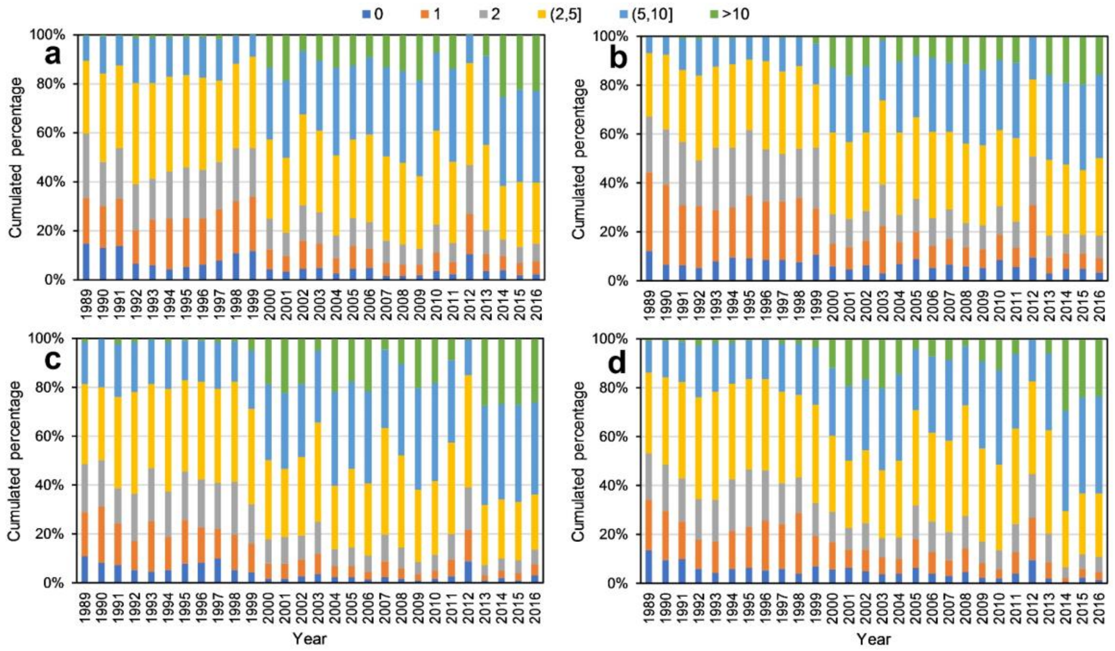

Supplementary Fig. 13 | Number of good observations in a season during 1989-2016 from Landsat images. a, Spring. **b**, Summer. **c**, Autumn. **d**, Winter.

Supplementary Figure. 14

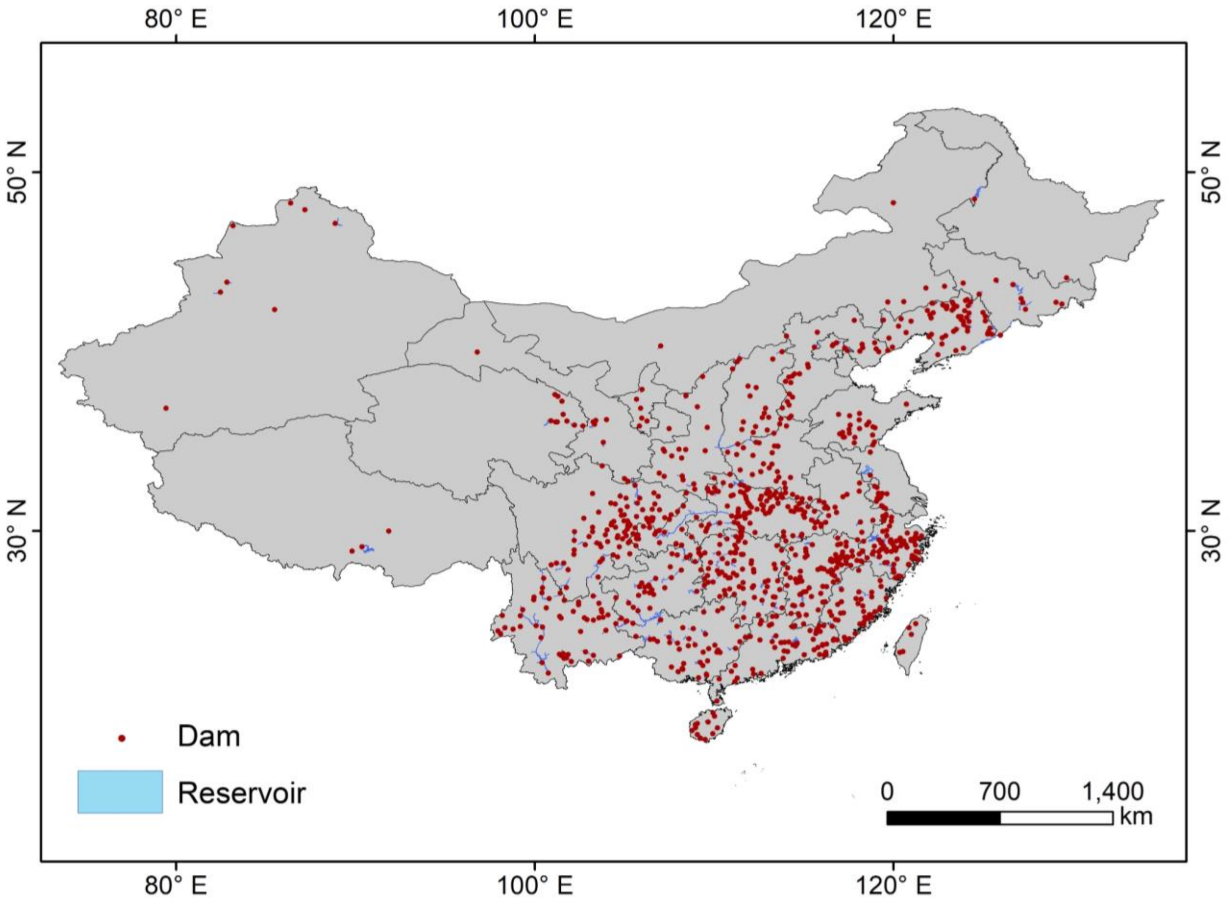

Supplementary Fig. 14 | Spatial distribution of dams and reservoirs in China from the Global Reservoir and Dam Database (GRanD) v1.3.

Supplementary Figure. 15

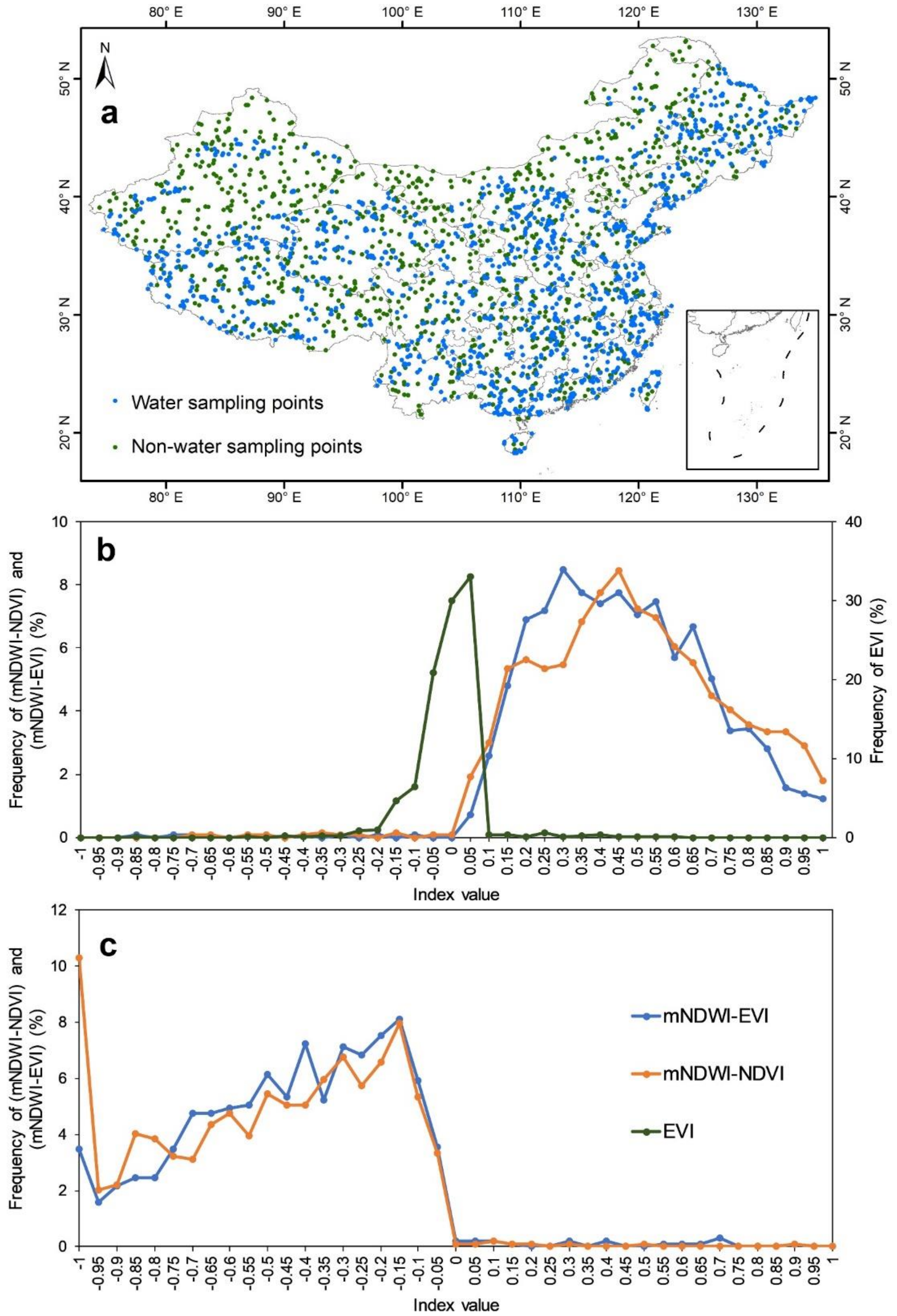

Supplementary Fig. 15 | Spectral characteristics of water and non-water sample points. a, Spatial distribution of sample points. **b,** mNDWI-EVI, mNDWI-NDVI, and EVI distributions of water sample points. **c,** mNDWI-EVI and mNDWI-NDVI distributions of non-water sample points.

Supplementary Figure. 16

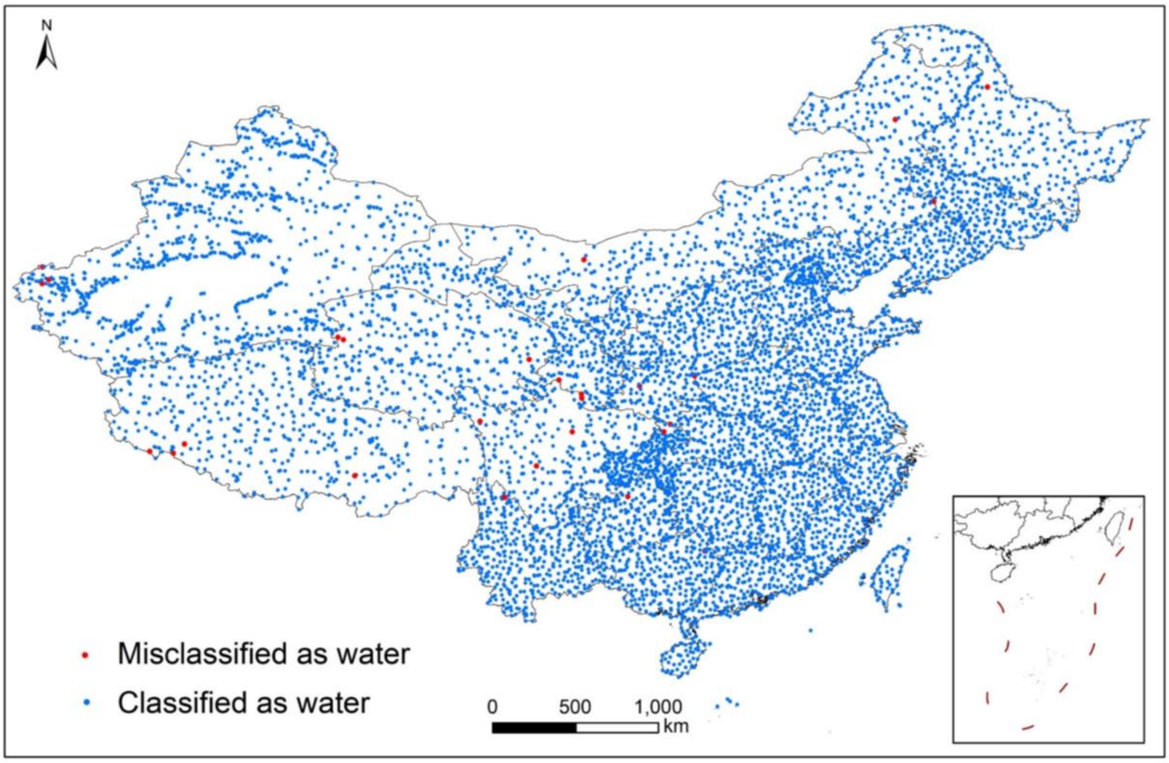

Supplementary Fig. 16 | Geographic distribution of validation sample points and error.

Supplementary Figure. 17

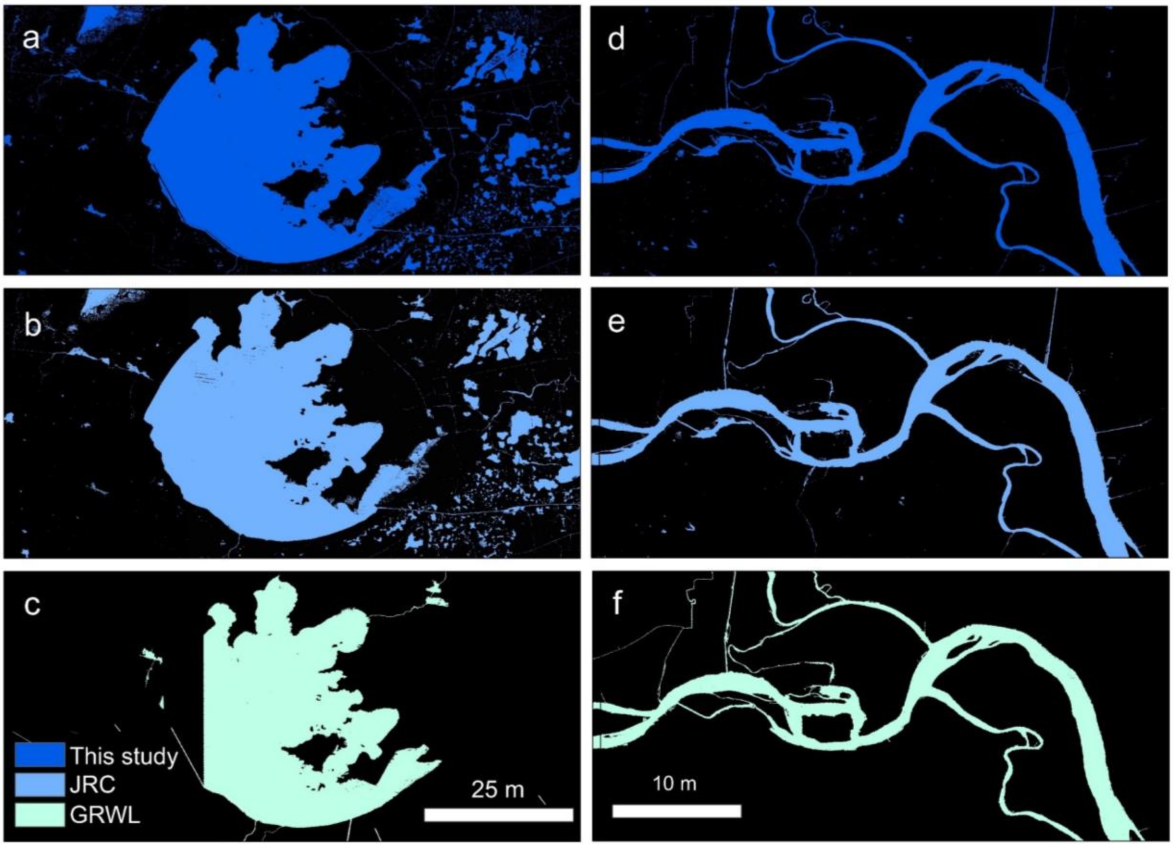

Supplementary Fig. 17 | Comparison of the JRC dataset, the Global River Widths from Landsat dataset (GRWL) dataset, and the surface water body dataset from this study. a, Taihu Lake from this study. **b**, Taihu Lake from the JRC dataset. **c**, Taihu Lake from the GRWL dataset. **d**, Yangtze River from this study. **e**, Yangtze River from the JRC dataset. **f**, Yangtze River from the GRWL dataset.

Supplementary Figure. 18

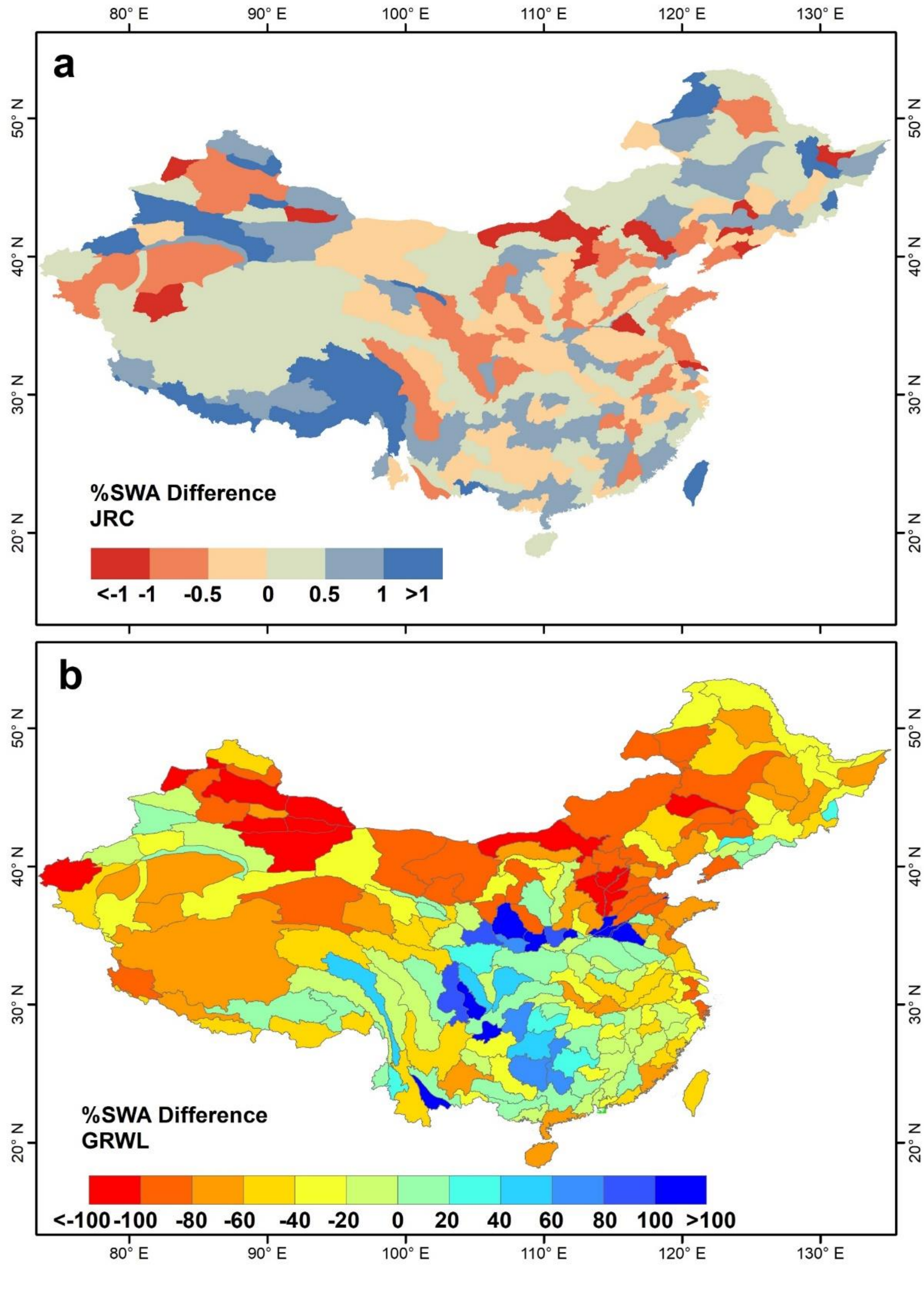

Supplementary Fig. 18 | Difference of surface water areas between this study and the JRC dataset (a) and the Global River Widths from Landsat dataset (GRWL) dataset (b) at the watershed scale.

Supplementary Figure. 19

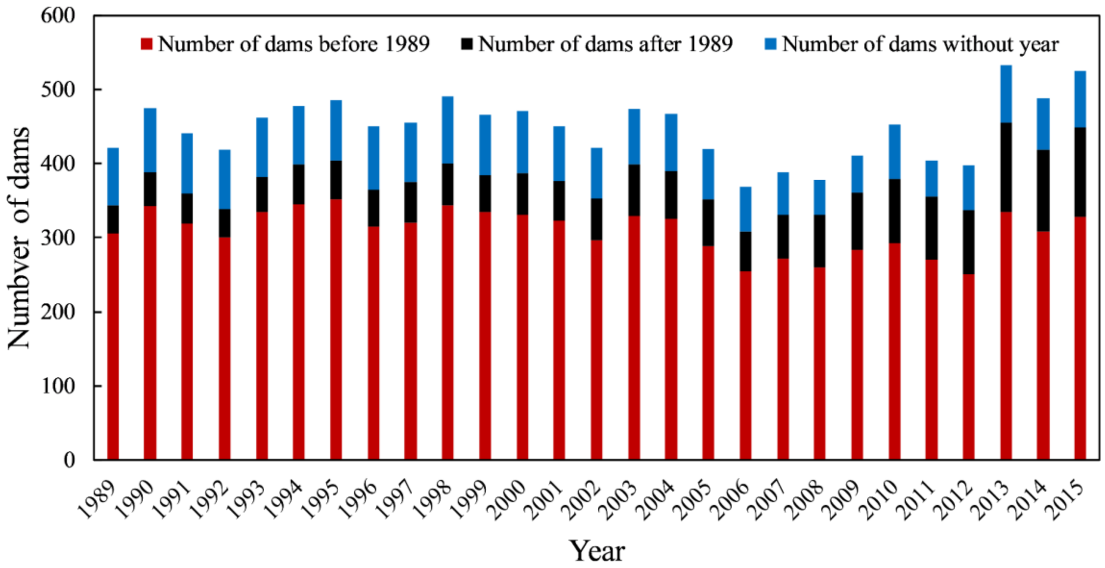

Supplementary Fig. 19 | Number of dams from the Global Reservoir and Dam Database (GRanD) v1.3 with different building years.

Supplementary Figure. 20

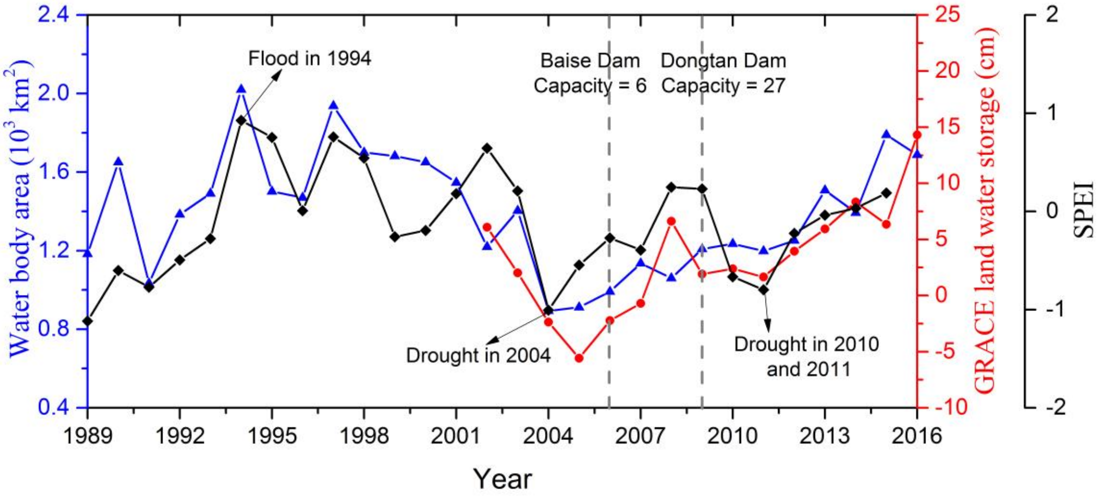

Supplementary Fig. 20 | Surface water area, terrestrial water storage (TWS), the standardized precipitation-evapotranspiration index (SPEI) from 1989 to 2016, and reservoir capacity in Guangxi Province.

Supplementary Figure. 21

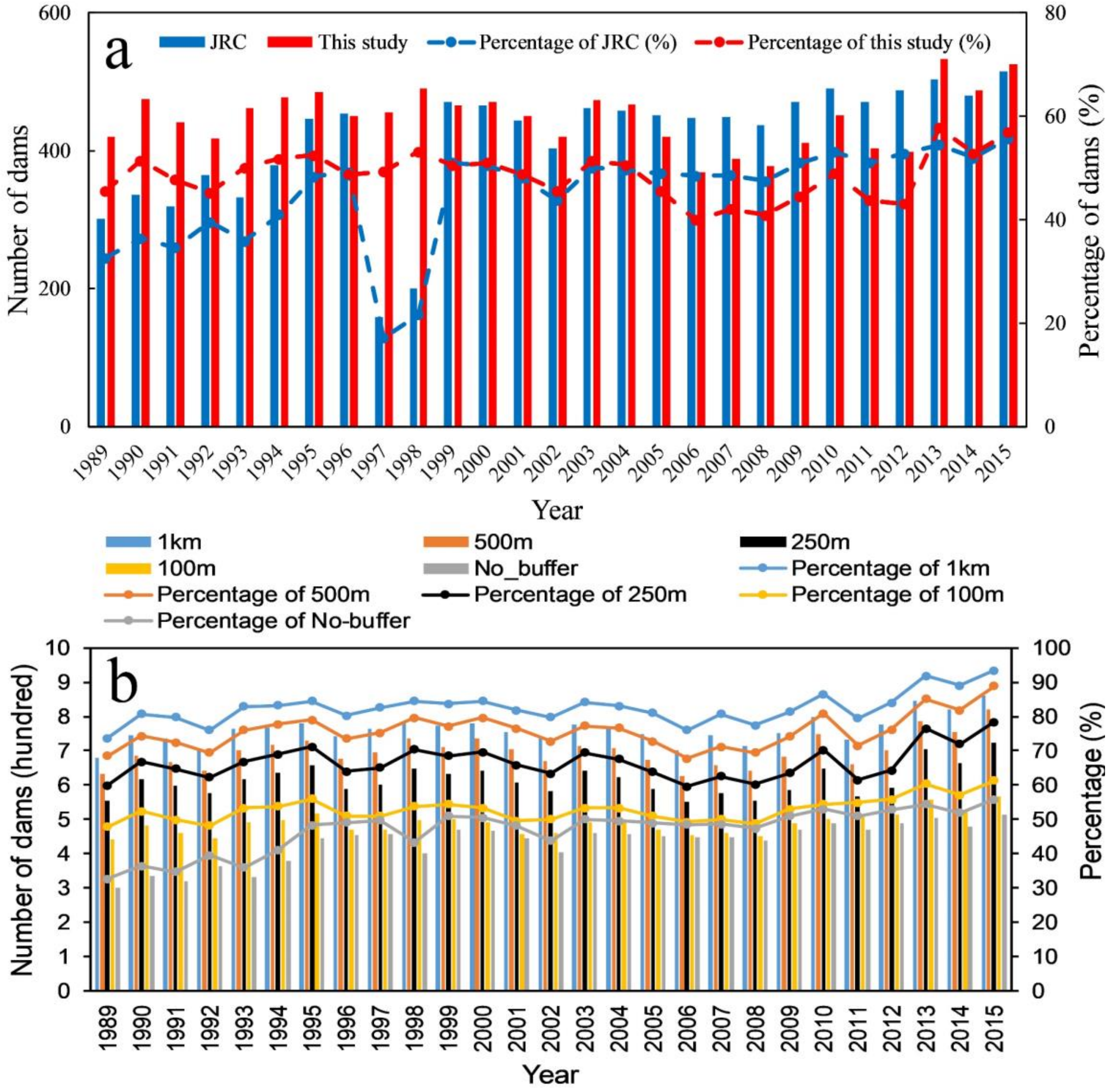

Supplementary Fig. 21 | Information on dams from the Global Reservoir and Dam Database (GRanD) v1.3. a, Number of dams intersected with our year-long surface water maps and the JRC dataset from 1989 to 2015. **b**, Number of dams intersected with our surface water maps using different buffer zones from 1989 to 2015.

Supplementary Figure. 22

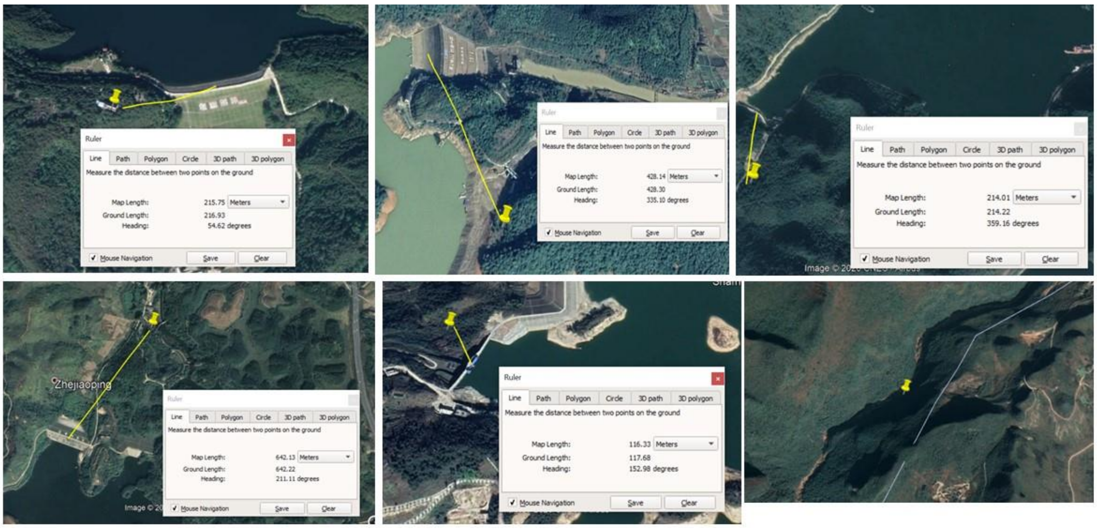

Supplementary Fig. 22 | Location errors of dams from the Global Reservoir and Dam Database (GRanD) v1.3.

Supplementary Table. 1

Supplementary Table 1. Consistent and divergent trends between terrestrial water storage (TWS) and surface water area (SWA) among 0.5° gridcells in China, as well as total human population (million) in those gridcells using Gridded Population of the World (GPW) of 2015. (Numbers in the Table are gridcell number / population number).

TWS vs SWA	SWA increase	SWA decrease	SWA no change	Total cells vs Total population
TWS increase	437 / 172	98 / 29	733 / 230	1268 / 431
TWS decrease	323 / 259	227 / 27	858 / 174	1408 / 460
TWS no change	236 / 107	84 / 53	658 / 266	978 / 426
Total cells / Total population	996 / 538	409 / 109	2249 / 670	3654 / 1317

Supplementary Table. 2

Supplementary Table 2. User's accuracy by province and seasonality class for the surface water body dataset in this study.

Year-long water					Seasonal water			
Province	Classified	Misclassified	Total	User's accuracy (%)	Classified	Misclassified	Total	User's accuracy (%)
Hebei	164	0	164	100.00	89	1	90	98.89
Shanxi	106	0	106	100.00	89	2	91	97.80
Shaaxi	168	1	169	99.41	69	1	70	98.57
Tibet	409	3	412	99.27	106	3	109	97.25
Sichuan	193	2	195	98.97	85	2	87	97.70
Zhejiang	91	0	91	100.00	46	1	47	97.87
Yunnan	266	1	267	99.63	137	3	140	97.86
Xinjiang	720	2	722	99.72	258	3	261	98.85
Qinghai	297	2	299	99.33	115	2	117	98.29
Gansu	295	3	298	98.99	56	2	58	96.55
Inner Mongolia	592	2	594	99.66	121	2	123	98.37
Heilongjiang	245	1	246	99.59	123	1	124	99.19
Jilin	194	1	195	99.49	72	1	73	98.63
Liaoning	162	0	162	100.00	62	0	62	100.00
Beijing	35	0	35	100.00	19	0	19	100.00
Tianjin	13	0	13	100.00	11	0	11	100.00
Ningxia	50	0	50	100.00	21	1	22	95.45
Shandong	136	0	136	100.00	81	2	83	97.59

Henan	134	1	135	99.26	84	1	85	98.82
Jiangsu	103	0	103	100.00	42	2	44	95.45
Anhui	95	0	95	100.00	87	0	87	100.00
Chongqing	156	1	157	99.36	61	2	63	96.83
Hubei	144	0	144	100.00	91	0	91	100.00
Hunan	138	1	139	99.28	114	1	115	99.13
Guizhou	112	1	113	99.12	94	1	95	98.95
Jiangxi	151	0	151	100.00	50	0	50	100.00
Guangxi	131	0	131	100.00	143	0	143	100.00
Guangdong	138	0	138	100.00	99	1	100	99.00
Fujian	88	0	88	100.00	73	1	74	98.65
Taiwan	34	0	34	100.00	25	0	25	100.00
Hainan	29	0	29	100.00	27	0	27	100.00
Total	5589	22	5611	99.71 (±0.12)	2550	36	2586	98.57 (±0.47)

Supplementary Table. 3

Supplementary Table 3. Producer's accuracy by province and by seasonality class for the surface water body dataset in this study.

Year-long water					Seasonal water			
Province	Classified	Misclassified	Total	Producer's accuracy (%)	Classified	Misclassified	Total	Producer's accuracy (%)
Hebei	101	0	101	100.00	92	2	94	97.87
Shanxi	116	0	116	100.00	39	4	43	90.70
Shaaxi	98	1	99	98.99	65	6	71	91.55
Tibet	854	11	865	98.73	402	41	443	90.74
Sichuan	295	3	298	98.99	109	21	130	83.85
Zhejiang	194	0	194	100.00	56	4	60	93.33
Yunnan	248	3	251	98.80	101	19	120	84.17
Xinjiang	294	8	302	97.35	220	31	251	87.65
Qinghai	310	6	316	98.10	157	36	193	81.35
Gansu	95	1	96	98.96	22	6	28	78.57
Inner Mongolia	656	3	659	99.54	181	25	206	87.86
Heilongjiang	493	4	497	99.20	208	19	227	91.63
Jilin	223	0	223	100.00	164	5	169	97.04
Liaoning	188	0	188	100.00	106	4	110	96.36
Beijing	18	1	19	94.74	4	6	10	40.00
Tianjin	21	0	21	100.00	11	1	12	91.67
Ningxia	34	1	35	97.14	14	2	16	87.50
Shandong	269	0	269	100.00	88	11	99	88.89

Henan	192	0	192	100.00	52	6	58	89.66
Jiangsu	167	0	167	100.00	80	8	88	90.91
Anhui	199	0	199	100.00	85	9	94	90.43
Chongqing	85	1	86	98.84	32	7	39	82.05
Hubei	214	0	214	100.00	132	17	149	88.59
Hunan	245	0	245	100.00	139	12	151	92.05
Guizhou	89	2	91	97.80	49	9	58	84.48
Jiangxi	215	1	216	99.54	88	16	104	84.62
Guangxi	222	1	223	99.55	119	21	140	85.00
Guangdong	201	1	202	99.50	191	16	207	92.27
Fujian	143	0	143	100.00	64	14	78	82.05
Taiwan	64	2	66	96.97	22	6	28	78.57
Hainan	81	0	81	100.00	39	11	50	78.00
Total	6624	50	6674	99.12 (±0.43)	3131	395	3526	86.43 (±3.57)

Supplementary Table. 4

Supplementary Table 4. Validation results of the surface water dataset in this study and the JRC dataset by seasonality class.

	Seasonal	Year-long		Seasonal	Year-long
User's accuracy of our study	98.57% (±0.47)	99.71% (±0.12)	Producer's accuracy of our study	86.43% (±3.57)	99.12% (±0.43)
User's accuracy of the JRC dataset	98.55%	99.60%	Producer's accuracy of the JRC dataset	68.44%	98.57%

Supplementary Note. 1

Supplementary Note 1. Effects of dams/reservoirs on surface water area and terrestrial water storage in Guangxi Province

Dam and reservoir construction is one of the main factors affecting the interannual surface water area (SWA) and terrestrial water storage (TWS) dynamics at the provincial scale[1,2]. One example is the Three Gorges Dam (TGD) in Hubei Province, the largest hydroelectric dam in the world. It has had large impacts on its neighboring provinces and watersheds along the Yangtze River since its first impoundment in 2003. Another example is Guangxi Province, which had two large important dams, the Baise Dam completed in 2006, and the Dongtan Dam completed in 2009 (https://en.wikipedia.org/wiki/List_of_dams_and_reservoirs_in_China
).

SWA and TWS in Guangxi Province had two stepwise increases after the severe drought in 2004 (Supplementary Fig. 20), corresponding well to the construction of these two large reservoirs. Before 2006, the interannual variations of SWA and TWS in Guangxi matched well with that of climate variation, which means that climate variability is the main factor affecting the interannual dynamics of SWA and TWS. Although Guangxi suffered from severe drought in 2010 and 2011, SWA and TWS didn’t decrease too much because of the regulation service of these large dams and reservoirs.

Supplementary Note. 2

Supplementary Note 2. Assessment of the accuracy of the geographical coordinates (longitude and latitude) for the location of dams in the GRanD v1.3

The Global Reservoir and Dam Database (GRanD) was first reported in 2011[3] and its late version (v1.3) was recently released (http://globaldamwatch.org/grand/). The data producers added a lot of dams and reservoirs in China, ranging from 62 dams in v1.1 to 923 dams in v1.3. The attributes of the GRanD dataset include dam name, geolocation, year the dams were constructed, and reservoir area. For this improved dataset, we first assessed the accuracy of the geographical coordinates (longitude and latitude) for the locations of dams. We overlaid the dams within our year-long surface water body maps and the JRC permanent surface water dataset from 1989-2015 (Supplementary Fig. 21a), and only there were average 448 ± 16, and 415 ± 33 dams out of 923 in China were intersected with our maps and the JRC dataset during 1989-2015, respectively. It means about 45%-49% of the GRanD dataset (923 dams in China) were found within both year-long surface water maps of our study and the JRC dataset. We further checked the dams with Google Earth images and found that the geographical coordinates of many dams had large errors larger than 500m (Supplementary Fig. 22). Then we created buffers with different distances (0, 100, 250, 500, 1,000m) based on these dams, and used these buffers to intersect with our surface water body maps to get the number of dams which intersected with our surface water maps (Supplementary Fig. 21b). About 761 ± 8 dams (82.4%) were intersected with the surface water maps when we used the buffer with the longest distance (1-km). Thus, these results suggested that the dam dataset still have great potential to be improved.

Supplementary References

1. Feng, L., Hu, C., Chen, X. & Zhao, X. Dramatic inundation changes of China's two largest freshwater lakes linked to the Three Gorges Dam. *Environ. Sci. Technol.* **47**, 9628–9634 (2013).

2. Fang, J. *et al.* Biodiversity changes in the lakes of the Central Yangtze. *Front. Ecol. Environ.* **4**, 369–377 (2006).

3. Lehner, B. *et al.* High-resolution mapping of the world's reservoirs and dams for sustainable river-flow management. *Front. Ecol. Environ.* **9**, 494–502 (2011).
